# Neomycin Interferes with Phosphatidylinositol-4,5-Bisphosphate at the Yeast Plasma Membrane and Activates the Cell Wall Integrity Pathway

**DOI:** 10.3390/ijms231911034

**Published:** 2022-09-20

**Authors:** Elena Jiménez-Gutiérrez, Teresa Fernández-Acero, Esmeralda Alonso-Rodríguez, María Molina, Humberto Martín

**Affiliations:** Departamento de Microbiología y Parasitología, Facultad de Farmacia, Instituto Ramón y Cajal de Investigaciones Sanitarias (IRYCIS), Universidad Complutense de Madrid, 28040 Madrid, Spain

**Keywords:** *Saccharomyces cerevisiae*, cell wall integrity, MAPK, Slt2, PIP_2_, neomycin

## Abstract

The cell wall integrity pathway (CWI) is a MAPK-mediated signaling route essential for yeast cell response to cell wall damage, regulating distinct aspects of fungal physiology. We have recently proven that the incorporation of a genetic circuit that operates as a signal amplifier into this pathway allows for the identification of novel elements involved in CWI signaling. Here, we show that the strong growth inhibition triggered by pathway hyperactivation in cells carrying the “Integrity Pathway Activation Circuit” (IPAC) also allows the easy identification of new stimuli. By using the IPAC, we have found various chemical agents that activate the CWI pathway, including the aminoglycoside neomycin. Cells lacking key components of this pathway are sensitive to this antibiotic, due to the disruption of signaling upon neomycin stimulation. Neomycin reduces both phosphatidylinositol-4,5-bisphosphate (PIP_2_) availability at the plasma membrane and myriocin-induced TORC2-dependent Ypk1 phosphorylation, suggesting a strong interference with plasma membrane homeostasis, specifically with PIP_2_. The neomycin-induced transcriptional profile involves not only genes related to stress and cell wall biogenesis, but also to amino acid metabolism, reflecting the action of this antibiotic on the yeast ribosome.

## 1. Introduction

Eukaryotic cells live in a constantly changing environment, so they need mechanisms to detect environmental signals and react to them in order to survive. Among these surveillance systems are the mitogen-activated protein kinase (MAPK) signaling pathways. These routes have a common structure based on cell surface sensors that trigger, through a GTPase switch, the activation of a p21-activated kinase (PAK) or protein kinase C (PKC). These active kinases, in turn, set off a three-tiered phosphorylation cascade composed of a MAP kinase kinase kinase (MAPKKK), a MAP kinase kinase (MAPKK), and a MAP kinase (MAPK), which finally conveys the signal to its effectors to elicit an adequate cell response to the stimulus detected [1]. The budding yeast *Saccharomyces cerevisiae* has five MAPKs immersed in five well-orchestrated signaling pathways that govern diverse cellular processes, such as the maintenance of intracellular osmolarity, mating, filamentous and invasive growth, spore wall assembly, and cell wall stability [2].

The cell wall integrity (CWI) pathway is primarily responsible for the preservation of this essential structure through cell wall synthesis and remodeling. The activation of this pathway is triggered not only by canonical cell wall alterations caused by exposure to cell wall-damaging agents, high or low temperature, changes in external osmolarity, or the presence of pheromones [3], but also by a variety of chemical agents and stress conditions not necessarily associated with the cell wall, such as rapamycin, alkaline pH, cadmium, actin-depolymerizing compounds, and plasma membrane, oxidative, or genotoxic stresses [4]. At the cell surface, CWI pathway-activating stimuli are perceived by a set of transmembrane mechanosensors, including Wsc1-3, Mid2, and Mtl1. They transmit the signal to the GTPase Rho1 via its guanosine nucleotide exchange factors (GEFs) Rom1/2, which interact with the plasma membrane (PM) phosphatidylinositol-4,5-bisphosphate (PIP_2_), and Tus1. Consequently, GTP-Rho1 activates Pkc1, responsible for the subsequent activation of the downstream Bck1-Mkk1/2-Slt2 MAPK module [3]. The phosphorylated and thus activated Slt2 MAPK, in turn, activates the transcription factor Rlm1, which modulates the expression of genes involved in the cell wall compensatory mechanism [5], and the SBF complex formed by Swi4 and Swi6, which regulates genes participating in cell cycle control [6] and a small set of cell wall-related genes [7].

Pkc1 is also positively regulated through phosphorylation by Pkh1/2 [8], homologs of the mammalian PM-associated 3-phosphoinositide-dependent protein kinase 1 (PDK1), and the target of rapamycin complex 2 (TORC2) [8,9]. On the other hand, TORC2 monitors the plasma membrane status and phosphorylates and activates protein kinases Ypk1/2 [10]. Although Ypk2 was first identified as a TORC2 substrate, subsequent work showed that Ypk1 plays a major role in downstream signaling to regulate multiple aspects of membrane physiology [10,11]. Ypk1, whose basal activity is conferred by Pkh1/2-mediated phosphorylation [12,13], regulates turgor pressure and plasma membrane tension [14]. Following the increase in plasma membrane tension, TORC2 signaling is activated via the pleckstrin homology (PH) domain adaptor proteins Slm1/2, by recruiting Ypk1 and Ypk2 to TORC2 for phosphorylation and activation [15]. It is very likely that there is some crosstalk between Ypk1- and Pkc1-dependent signaling, as Ypk1 regulates plasma membrane homeostasis and composition, and the localization of different components of the CWI pathway, such as Rom2 or Rho1, depends on plasma membrane components [16].

The lack of a fine-tuned performance from MAPK pathways leads to disastrous outcomes, such as cell death in yeast, and cancer or inflammatory or neurodegenerative disorders in humans [17]. There are regulatory mechanisms, such as protein phosphatases or negative feedback loops, to control the magnitude and timing of the response [18,19]. Nevertheless, signal amplification through positive feedback loops has also been described [20], providing phenomena such as bistable switches [21]. By using a synthetic biology approach, our group generated a genetic positive feedback loop in the CWI pathway consisting of a plasmid bearing the hyperactive allele of one of the MAPKKs, *MKK1^S386P^* [22], placed under the control of the *MLP1* promoter, one of the most induced genes regulated by the transcription factor Rlm1 [23]. Triggering this artificial circuit with a cell wall insult results in cell death due to hyperactivation of the CWI pathway. This so-called Integrity Pathway Activation Circuit (IPAC) proved to be a powerful genetic tool for the discovery of novel signaling regulators of this pathway [24].

In this study, by integrating the IPAC into the *S. cerevisiae* genome, we generated a yeast strain with increased sensitivity to cell wall stress, used to screen a collection of compounds in the search for novel stimuli. This IPAC-integrated strain enabled us to identify five new CWI pathway-activating compounds, namely the aminoglycoside neomycin, lithium chloride (LiCl), zinc chloride (ZnCl_2_), ethylenediaminetetraacetic acid (EDTA), and the antihistamine diphenhydramine (DPH). We further investigated the contribution of the CWI pathway and the transcriptional profiling of the yeast cell response to neomycin, as this aminoglycoside activates the MAPK Slt2 in contrast to other protein synthesis inhibitors assayed.

## 2. Results

### 2.1. Genomic Integration of IPAC Drives Higher CWI Pathway Activation Than Its Expression from a Centromeric Plasmid

**Figure 1:**
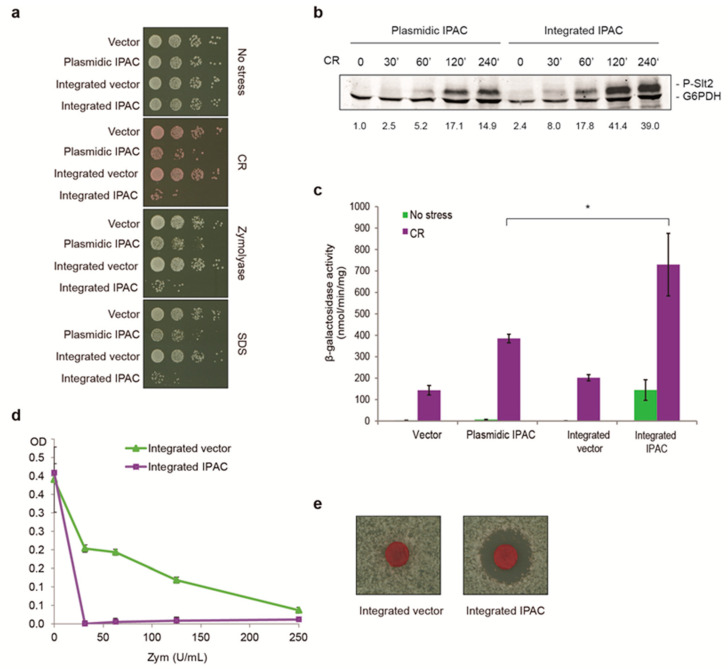
Integration of IPAC results in greater CWI pathway hyperactivation than the IPAC-bearing plasmid. (**a**) Sensitivity to different stresses of BY4741 strain bearing either the empty vector YCplac111 or the plasmid YCplac111-IPAC, and control wild type with the integrated vector (YSTH1) or with integrated IPAC (YSTH2). Ten-fold serial dilutions of cell suspensions were spotted onto YPD plates in the absence (no stress) or presence of 5 μg/mL CR, 10 U/mL (100 μg/mL) zymolyase 100T, or 100 μg/mL SDS. A representative assay from three different experiments with distinct transformants is shown. (**b**) Western blotting analysis of extracts from BY4741 strain transformed with plasmid YCplac111-IPAC or the YSTH2 strain (integrated IPAC) treated with 30 μg/mL CR for the indicated times (min). Dually phosphorylated Slt2 and G6PDH (as a loading control) were detected with anti-phospho-p44/42 and anti-G6PDH antibodies, respectively. The numbers below correspond to the amount of phosphorylated Slt2, normalized with respect to the loading control for each sample and expressed as a fold increase relative to the basal phosphorylation levels in the absence of stress of BY4741 cells bearing the YCplac111-IPAC plasmid. A representative blot from three independent experiments is shown. (**c**) β-galactosidase activity of cell extracts from the same strains as in (**a**), all bearing the *MLP1*p-*lacZ* plasmid. Cells were either left untreated (no stress) or treated for 4 h with 30 μg/mL CR. Data represent the mean of β-galactosidase activity of three independent transformants. Error bars indicate standard deviation, and an asterisk indicates statistical significance relative to plasmidic IPAC according to Student’s *t*-test (* = *p*-value < 0.05). (**d**) Multiwell plate sensitivity assay of the YSTH1 strain (integrated vector) and the YSTH2 strain (integrated IPAC) to the indicated concentrations of zymolyase 100T. Data represent the mean of three independent transformants. Error bars indicate standard deviation. (**e**) Halo sensitivity assay of the same strains as in (**d**). Disks were impregnated with 10 mg/mL CR. A representative assay from three different experiments is displayed.

In order to improve the performance of the positive feedback circuit IPAC [24] as a screening tool, we explored the effect of its integration into the yeast genome by using different constructs in several genetic backgrounds. As shown in Figure 1a, we found that integration of IPAC at the *HO-SSB1* locus in strain Y3656 promoted greater growth inhibition under different CWI pathway stimulating conditions (Congo red (CR), zymolyase, and SDS) than its expression from a centromeric plasmid. Although both IPAC versions elicited similar Slt2 activation kinetics, the level of Slt2 phosphorylation in yeast cells with integrated IPAC in the presence of CR was almost triple that found in cells bearing the plasmid version (Figure 1b). This increase in IPAC-induced CWI pathway activation in IPAC-integrated yeast cells was also observed at the transcriptional level. In Figure 1c, the *MLP1*p-*LacZ*-based transcriptional assay [25] showed higher *MLP1* expression levels (approximately 2-fold) in the case of the integrated IPAC in comparison with the plasmid IPAC version when cells were treated with CR. These results confirmed that the greater sensitivity to cell wall stress caused by integration of the IPAC was due to a stronger hyperactivation of the CWI pathway. Not only the hypersensitivity, but also the stability of the IPAC integrated in the genome that allows the use of complete media, are advantages over the plasmid versions in the search for novel CWI stimuli.

As shown in Figure 1, the strain with integrated IPAC can be used in different liquid or solid media formats; it always displays higher sensitivity to canonical cell wall perturbing agents than the control strain without this circuit, as observed for zymolyase in a multiwell plate (Figure 1d) or CR in halo (Figure 1e) assays.

### 2.2. Identification of Novel CWI Pathway-Activating Compounds by Using the IPAC

**Figure 2:**
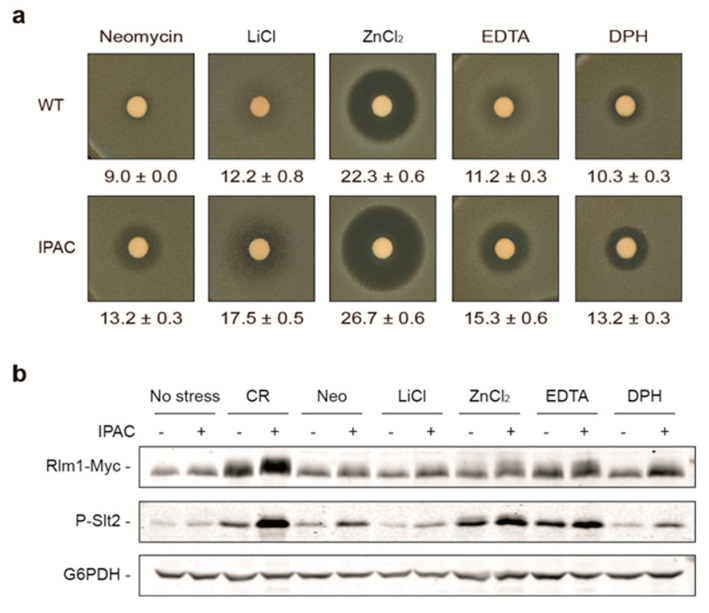
Neomycin, lithium chloride (LiCl), zinc chloride (ZnCl2), ethylenediaminetetraacetic acid (EDTA), and diphenhydramine chlorhydrate (DPH) activate the IPAC circuit. (**a**) Growth inhibition halo assay of vector (WT) or IPAC-integrated Y3656 strains. Six-millimeter-diameter disks were impregnated with 50 mg/mL neomycin sulfate, 424 mg/mL LiCl, 136 mg/mL ZnCl_2_, 11 mg/mL EDTA, or 50 mg/mL DPH. Mean and standard deviations of halo diameters from three different experiments are shown. (**b**) Western blotting analysis of extracts from BY4741 RLM1-6MYC strain transformed with YCplac111 (−) or Ycplac111-IPAC (+) and treated or not (no stress) for 3 h with 30 μg/mL CR, 10 mg/mL neomycin (Neo), 8.5 mg/mL LiCl, 681.4 μg/mL ZnCl_2_, 11.2 mg/mL EDTA, or 1 mg/mL DPH. Rlm1-Myc, dually phosphorylated Slt2, and G6PDH (as a loading control) were detected with anti-Myc, anti-phospho-p44/42, and anti-G6PDH antibodies, respectively. A representative blot from two independent experiments is shown.

Subsequently, we screened 94 diverse compounds (Table S1) on the strain with integrated IPAC and its corresponding wild type by performing halo sensitivity assays. Only five generated larger growth inhibition halo in cells containing IPAC than in cells without IPAC. Among these compounds were the aminoglycoside neomycin, lithium chloride (LiCl), zinc chloride (ZnCl_2_), ethylenediaminetetraacetic acid (EDTA), and the antihistamine diphenhydramine (DPH) (Figure 2a). As expected, phospho-Slt2 levels induced by these compounds were also more prominent in combination with the IPAC than in its absence. This was concomitant with an increase in the amount and mobility shift of Rlm1 (Figure 2b), meaning that this transcription factor is undergoing activating phosphorylation by Slt2 [26], which reflects that the IPAC feedback loop is functioning and thus causing the hyperactivation of the CWI pathway.

### 2.3. Neomycin, but Not Other Aminoglucosides, Activates the CWI Pathway

**Figure 3:**
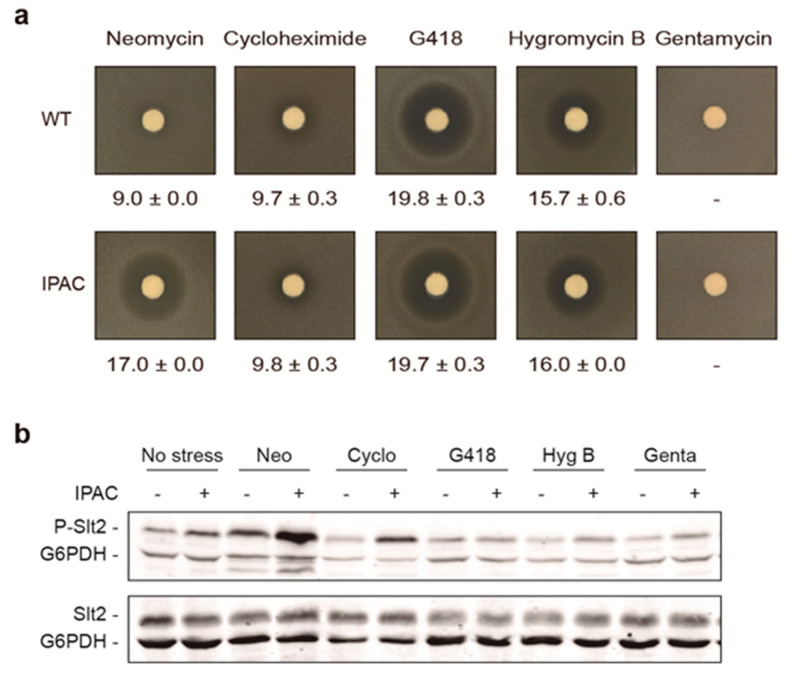
Neomycin is the only protein synthesis inhibitor that activates the CWI pathway. (**a**) Halo sensitivity assay of vector (WT) or Y3656 strains with integrated IPAC. Disks were impregnated with 100 mg/mL of neomycin sulfate, 1 mg/mL of cycloheximide, 50 mg/mL of G418, 50 mg/mL of hygromycin B, or 50 mg/mL of gentamycin. Mean and standard deviations of halo diameters from three different experiments are shown. (**b**) Western blotting analysis of extracts from Y3656 strain without (−) or with IPAC (+) and treated or not (no stress) for 4 h with 10 mg/mL neomycin, 25 μg/mL cycloheximide, 31.3 μg/mL G418, 62.5 μg/mL hygromycin B, or 10 mg/mL gentamycin sulfate. Dually phosphorylated Slt2, total Slt2 protein, and G6PDH (as a loading control) were detected with anti-phospho-p44/42, anti-Slt2, and anti-G6PDH antibodies, respectively. A representative blot from three independent experiments is shown.

Of the compounds tested, there were a few aminoglycosides (kanamycin, gentamycin, G418, and neomycin) (Table S1), but only neomycin turned on the IPAC circuit. Thus, to investigate whether this outcome was specific to this drug, we analyzed by halo assay the effect on IPAC activation of higher concentrations of gentamycin (50 mg/mL), G418 (50 mg/mL), and neomycin (100 mg/mL) than those used in the screening (10 mg/mL), as well as another aminoglycoside, hygromycin B (50 mg/mL), and cycloheximide (1 mg/mL), a non-aminoglycoside protein synthesis inhibitor widely used in yeast studies [27]. As shown in Figure 3a, neomycin was again the only one that generated a growth inhibition halo of larger diameter in yeast cells carrying the IPAC than in cells without it. In these latter cells, the activity of this antibacterial aminoglycoside was practically negligible, as expected. Gentamycin, which, as with neomycin, has only antibacterial activity, did not inhibit the growth of either strain, while aminoglycosides hygromycin B and G418, known for their activity in both bacterial and eukaryotic cells, caused similar inhibition halos of considerable diameter in yeast cells irrespective of the presence of IPAC. Lastly, sensitivity to cycloheximide was moderate and similar in cells with or without IPAC.

To assess the activation of the CWI pathway following treatment with these compounds, Slt2 phosphorylation was analyzed by Western blotting. While neomycin induced phosphorylation of this MAPK relative to the untreated control both in the presence and absence of IPAC, cycloheximide only increased phospho-Slt2 levels in combination with IPAC, whereas gentamycin, G418, and hygromycin B treatments did not promote the activation of Slt2 at all (Figure 3b). To rule out the possibility that the lack of Slt2 phosphorylation observed in the presence of the latter compounds was a consequence of protein synthesis inhibition, Slt2 protein levels were also analyzed, with no apparent decrease in the amount of MAPK (Figure 3b).

### 2.4. Analysis of Signaling Triggered by Neomycin through the Cell Wall Integrity Pathway

**Figure 4:**
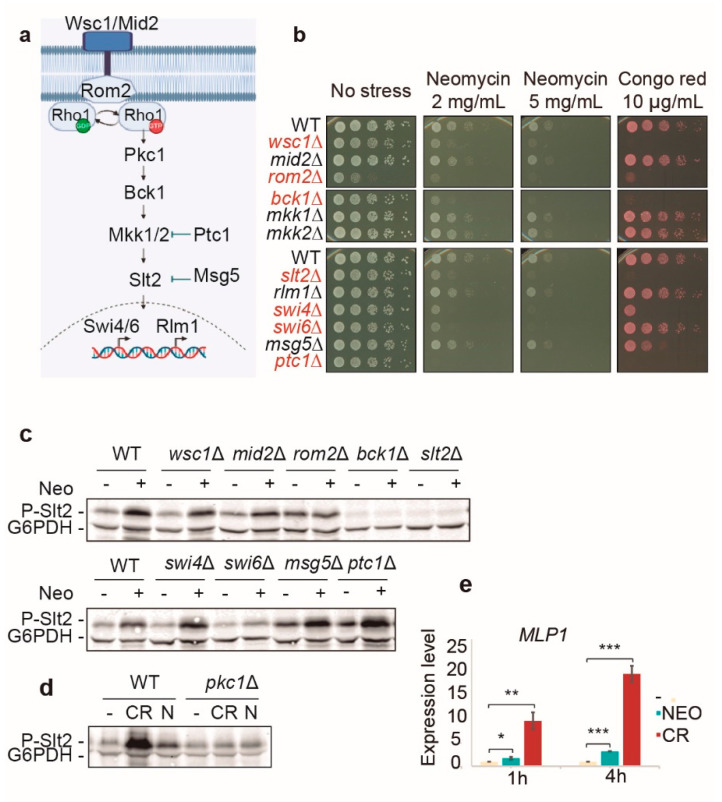
The CWI pathway is essential for yeast cells to survive in the presence of neomycin. (**a**) Scheme depicting the main components of the CWI pathway. (**b**) Sensitivity to neomycin of isogenic Y3656 mutants lacking the indicated CWI pathway components was assayed by spotting 10-fold serial dilutions of cell suspensions onto YPD plates in the absence (no stress) or presence of either 2 or 5 mg/mL neomycin or 10 μg/mL of CR. Red letters indicate higher sensitivity to neomycin than the wild-type strain. A representative assay from three different experiments is shown. (**c**) Neomycin-elicited signaling to Slt2 is mediated by core components of the CWI pathway. Western blotting analysis of extracts of isogenic Y3656 mutants lacking the indicated CWI pathway components treated (+) or not (−) for 4 h with 10 mg/mL neomycin. (**d**) Western blotting analysis of CML128 (WT) and *pkc1*Δ mutant isogenic strains in the absence (−) or presence of 30 μg/mL CR or 10 mg/mL neomycin (N) for 4 h. Dually phosphorylated Slt2 and G6PDH (as a loading control) were detected with anti-phospho-p44/42 and anti-G6PDH antibodies, respectively. A representative blot from three independent experiments is displayed. (**e**) Neomycin induces Rlm1-mediated gene transcription. Transcriptional induction analysis of the *MLP1* gene by RT-qPCR in response to neomycin compared to CR in the Y3656 strain. Cells were cultured in the absence of stress (−) or with 10 mg/mL neomycin or 30 μg/mL CR for 1 or 4 h. The graph shows the mean and standard deviation of the expression level from three independent experiments. Asterisks indicate statistical significance relative to the absence of stress according to a Student’s *t*-test (* = *p*-value < 0.05; ** = *p*-value < 0.01; *** = *p*-value < 0.001).

Next, we wanted to know the role of the CWI pathway in the response of yeast cells to neomycin. To this end, we tested the sensitivity to this drug of mutants lacking different components of this signaling pathway (Figure 4a). We could not use the Euroscarf collection of nonessential gene mutants because gene disruptions were made by inserting the kanamycin resistance gene, resulting in cross-resistance to neomycin. Instead, the mutants were obtained from a subcollection generated in the Y3656 background using the nourseothricin resistance gene [28].

Figure 4b shows that the absence of key elements of the CWI pathway, such as the mechanosensor Wsc1, the Rho1 GEF Rom2, the MAPKKK Bck1, the MAPKK Slt2, and the SBF transcription factor components Swi4 and Swi6, increased the sensitivity of cells to neomycin. This means that the CWI pathway is essential for yeast survival in the presence of this aminoglycoside. In addition, the pattern of sensitivity to this compound closely resembles that of CR except for the *swi6*Δ mutant, which was very sensitive to neomycin but grew similar to the wild-type strain in the presence of CR. As for phosphatases, the absence of Ptc1, which negatively regulates Mkk1/2, was deleterious in the presence of both neomycin and CR. In contrast, the lack of the dual-specificity phosphatase Msg5 had minimal impact on growth with neomycin but led to reduced growth with CR. These results are consistent with the hyperactivation of the CWI pathway in both *ptc1*Δ and *msg5*Δ mutants and the much higher sensitivity to cell wall stress already observed in the former [29,30].

When we analyzed the phospho-Slt2 levels of these mutants treated with neomycin, only the *rom2*Δ, *bck1*Δ, and *swi6*Δ mutants showed no increase in Slt2 phosphorylation after treatment, reflecting the involvement of these components in signal transmission through the CWI pathway in response to neomycin (Figure 4c). The kinase Pkc1, responsible for the activation of the MAPK module, was also necessary to transmit the neomycin-induced signaling to Slt2, as happens with CR (Figure 4d). In contrast, the *wsc1*Δ, *swi4*Δ, and *ptc1*Δ mutants displayed a rise in phospho-Slt2 levels after neomycin exposure (Figure 4c) despite being quite sensitive to this drug (Figure 4b). Therefore, it seems that a functional CWI pathway is essential for yeast cells to survive neomycin exposure.

We also wanted to know if Rlm1 was active and regulating the canonical CWI gene response under these conditions. To do so, we measured by RT-qPCR the expression levels of *MLP1*, which is one the most induced genes under cell wall stress conditions. As shown in Figure 4e, there was a significant induction of this Rlm1-regulated gene after neomycin exposure, although much lower than the one triggered by CR.

### 2.5. Neomycin Reduces PIP_2_ Availability at the Yeast Plasma Membrane

**Figure 5:**
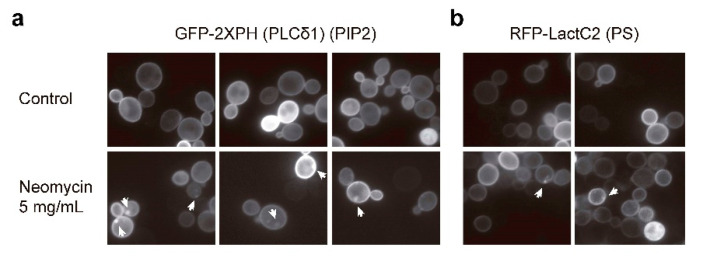

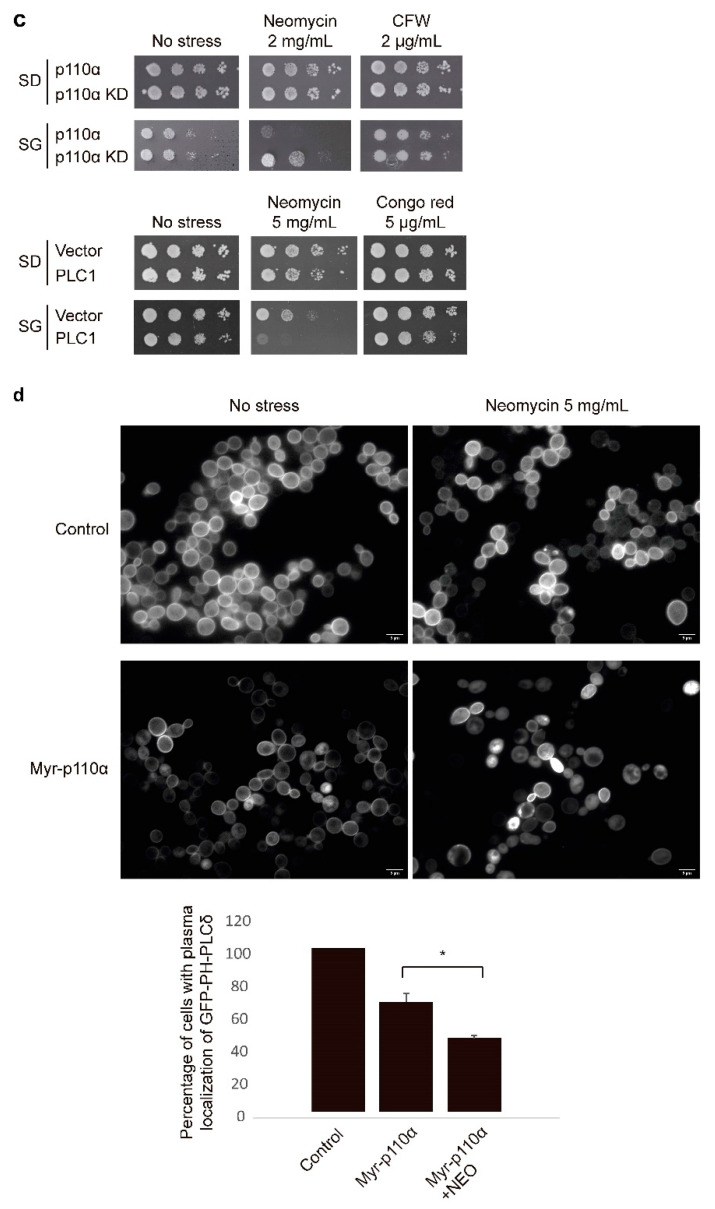
(**a**) Fluorescence microscopy images of the wild-type YPH499 strain co-expressing the PIP_2_ marker GFP-2XPH (PLCδ) and p110α-KD from the plasmids pJMCS-DM23 and YCpLG-p110αKD, respectively, treated or not with neomycin 5 mg/mL and cultured in SR-G for 5 h. (**b**). Fluorescence microscopy images of YPH499 strain expressing the phosphatidylserine (PS) marker RFP-LactC2 from the plasmid pRS416- RFP-LactC2 treated or not with neomycin 5 mg/mL cultured in SD for 5 h. (**c**) Growth assays of YPH499 strain transformed with plasmids YcpLG-p110α or YcpLG-p110αKD on SD and SG plates containing no stress, 2 mg/mL of neomycin, or 5 µg/mL of CFW (upper panel). Growth assays of YPH499 cells transformed with plasmids BG1805-PLC1 or pYES2 as control on SD/SG plates containing no stress, 5 mg/mL of neomycin, or 10 µg/mL of CR (lower panel). A representative assay from three independent experiments is shown. (**d**) Fluorescence microscopy images of the YPH499 strain expressing the PIP_2_ marker GFP-2X-PH(PLCδ1) with either an inactive version of p110α (control) or Myr-p110α from plasmids pJMCS-DM23, YcpLG-p110αKD and YcpLG-Myr-p110α, respectively, treated or not with 5 mg/mL of neomycin for 5 h and cultured in SR-G. Scale bars correspond to 5 μm. The graph shows the mean percentage of cells with PM labeling with GFP-2XPH (PLCδ1) from three independent experiments and error bars indicate the standard deviation. An asterisk indicates statistical significance relative to the control strain according to a Student’s *t*-test (* = *p*-value < 0.05).

Neomycin has been used as a PIP_2_-sequestering agent [31,32,33] due to the high-affinity electrostatic interaction between its positively charged moiety and the negative charge of this lipid. We wondered if the effects we observed in yeast cells upon neomycin treatment were due to its interference with PIP_2_ at the PM. Indeed, previous studies carried out by our group showed that PIP_2_ elimination by overexpression of a PM-directed version of the catalytic subunit of mammalian PI3K p110α-CAAX caused activation of the CWI pathway and growth inhibition due to the essential role of this lipid in yeast physiology [34]. Accordingly, we first microscopically tracked the presence of PIP_2_ with a fluorescent reporter consisting of two tandem copies of the pleckstrin homology (PH) domain of the phospholipase C δ1 (PH-PLC δ1) fused to GFP. As shown in Figure 5a, the PIP_2_ reporter was present at the PM to the same extent in neomycin-treated and untreated cells. However, there were some spots that seemed to be associated with PM, which were observed in cells exposed to neomycin (in 40.77 ± 0.054% cells) and absent in untreated control cells. These spots also appeared when the presence of phosphatidylserine, another plasma membrane lipid, was detected with the specific fluorescent reporter RFP-LactC2 (Figure 5b).

This result led us to think that the binding of PH-PLC δ1 probe to PIP_2_ competed with that of the neomycin. In fact, other authors previously demonstrated in mammalian cells that neomycin blocked the hydrolytic activity of PLC on PIP_2_, whereas in the presence of PH-PLC δ1-GFP 2, PLC was no longer susceptible to neomycin blockade [35]. Thus, we decided to check by other means if accessibility to PIP_2_ was compromised by neomycin. To do so, we analyzed whether the growth inhibition caused by the elimination of PIP_2_ was enhanced in the presence of neomycin. As mentioned above, the expression of a version of mammalian p110α targeted to the PM by a CAAX prenylation signal leads to drastic PIP_2_ depletion that causes strong growth inhibition. However, the soluble version of p110α leads to lower levels of conversion of PIP_2_ without compromising cell growth [36,37].

As shown in Figure 5c, upon galactose induction, neomycin toxicity was enhanced as compared to a glucose-based medium (SD), probably due to the effect of this carbon source on cell wall metabolism. Remarkably, exposure to neomycin enhanced the growth inhibition of p110α-expressing cells but did not affect the growth of cells expressing a p110α kinase-dead mutant. This effect seemed to be specific to neomycin, as it did not happen with calcofluor white. Similar results were observed when cells overexpressed phospholipase C1 (PLC1), which catalyzes the hydrolysis of PIP_2_ to generate inositol 1,4,5-triphosphate (IP3) and 1,2-diacylglycerol (DAG) (Figure 5c). Thus, neomycin potentiates the growth inhibition derived from PIP_2_ elimination by both PI3K and PLC1.

Therefore, we thought we might be able to observe the effects of neomycin on the GFP-PH-PLC δ1 localization in cells in which the levels of PIP_2_ were already diminished. To this end we used the PI3K version Myr-p110α, which is targeted to the PM by a myristoylation signal and possesses an intermediate PIP_2_-converting activity when expressed in yeast cells [36]. Consistently, the percentage of Myr-p110α cells with GFP-PH-PLC δ1 at the PM was lower than that of control cells devoid of PI3K activity. After neomycin treatment, the percentage of cells expressing Myr-p110α with the PIP_2_ reporter at the PM was significantly reduced compared to untreated cells (Figure 5d), meaning that neomycin might be binding to PIP_2_ and thus preventing labelling with the reporter. These results indicate that neomycin might reduce the availability of this lipid at the yeast cell PM, thus leading to CWI pathway activation. Indeed, although treatment of wild-type cells with neomycin did not result in significant alteration of chitin content in the yeast cell wall or a change in the axial budding pattern, it induced a modest increase in cell lysis in a dose-dependent manner (Supplementary Figure S1).

### 2.6. TORC2-Dependent Ypk1 Phosphorylation Is Reduced upon Neomycin Exposure

**Figure 6:**
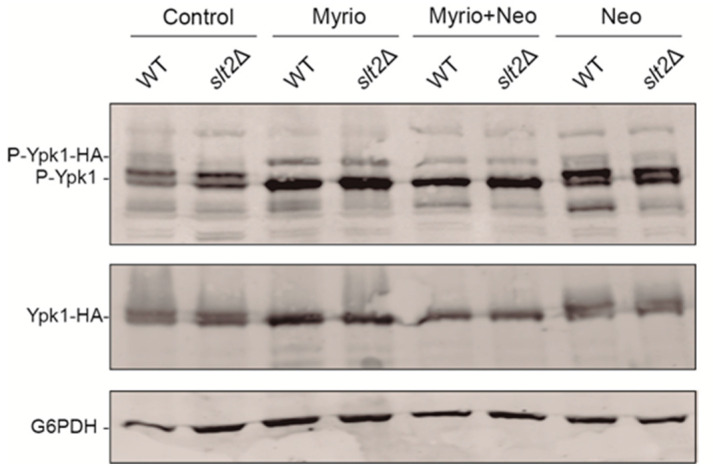
Western blotting of cell lysates of Y3656 and the isogenic *slt2*∆ strains transformed with plasmid pLB215 (Ypk1-HA) from untreated cultures (control) or treated with 0.8 mg/mL (2 µM) myriocin for 2 h (Myrio), the combination of 0.8 mg/mL (2 µM) and 5 mg/mL neomycin for 2 h (Myrio+Neo), or 5 mg/mL neomycin for 4 h (Neo), all of them cultured in YPD. Membranes were probed with anti-phospho T662-Ypk1 (upper panel), anti-HA (middle panel), and anti-G6PDH (lower panel), as a loading control.

The Slm1–TORC2–Ypk1 pathway is required for PM lipid homeostasis in yeast cells. The central protein in this route is the kinase Ypk1, which, in response to lipid alterations, is recruited to the PM by Slm1 for phosphorylation by TORC2 and subsequently by Pkh1/2 in order to activate several downstream proteins regulating complex lipid metabolism. Interestingly, Slm1 contains a PH domain specific to PIP_2_ that is crucial for the recruitment of Ypk1 to the PM [10]. We decided to test whether the restriction of PIP_2_ availability caused by neomycin altered the activation of this pathway. To this end, we analyzed TORC2-dependent Ypk1 phosphorylation on a HA tagged version expressed from a centromeric plasmid, and on the endogenous one by using a phospho-specific antibody directed against the phosphorylated T662 of Ypk1. As a positive control for Ypk1 phosphorylation, we used cells treated with myriocin, a compound that inhibits sphingolipid biosynthesis. As expected, cells treated with myriocin showed phosphorylation at the T662 of both the plasmid and endogenous version of Ypk1 (Figure 6). In contrast, neomycin treatment did not cause phosphorylation of Ypk1. Moreover, it reduced the phosphorylation of Ypk1 in cells simultaneously treated with myriocin, suggesting that TORC2-mediated signaling is inhibited by neomycin treatment. Therefore, neomycin could be either hindering Slm1 binding to PIP_2_ at the PM and subsequent recruitment of Ypk1, or directly affecting TORC2 function.

One of the subunits of the TORC2 complex, Avo2, has been identified as a phosphorylation substrate of the MAPK Slt2, which downregulates TORC2 function under cell wall stress conditions [38]. If that were the case in the presence of neomycin, an increase in Ypk1 phosphorylation should occur in the absence of Slt2. As shown in Figure 6, Ypk1 phosphorylation levels were similar in the wild-type and *slt2*∆ cells upon treatment with myriocin, neomycin, or a combination of both. Thus, the mechanism by which neomycin causes a reduction in TORC2-dependent Ypk1 phosphorylation is not a direct TORC2 downregulation mediated by Slt2, but rather an indirect effect, likely due to reduced Slm1-dependent Ypk1 recruitment at the PM.

### 2.7. Transcriptional Profile Induced by Neomycin

**Figure 7:**
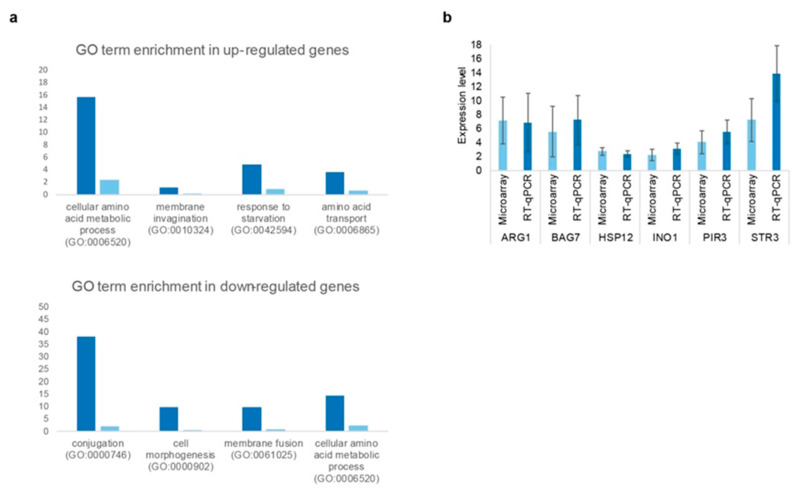
Genome-wide expression profile of wild-type cells following neomycin treatment. (**a**) Gene Ontology term enrichment within the genes up- or downregulated upon neomycin exposure. The graph indicates the percentage of genes clustered into the indicated GO terms for the differentially expressed genes upon neomycin treatment (dark blue) compared to that of the overall genome (light blue). According to a χ2 test, only *p*-values ≤ 0.05 were considered. (**b**) Comparison of the neomycin transcriptional induction determined by DNA microarrays and RT-qPCR. The graphic shows the level of induction of six genes selected to confirm by RT-qPCR (dark blue) the initial values obtained in the DNA microarray assays (light blue). The columns represent the mean, and the error bars indicate the standard deviation from three independent experiments.

We performed a DNA microarray analysis to determine the transcriptional profile of cell response to neomycin. Upon the treatment of yeast cells with this aminoglycoside, 83 genes were induced (≥1.7 fold) and 21 were repressed (≤0.6 fold) compared to untreated control cells (Tables S2 and S3). We then performed a Gene Ontology (GO) analysis by which we observed a significant enrichment of upregulated genes in the functional groups corresponding to amino acid metabolism, amino acid transport, starvation response and membrane invagination, while the downregulated genes were mostly involved in conjugation (Figure 7a and Tables S4 and S5). To verify the validity of the transcriptional profiling data obtained with neomycin, we performed RT-qPCRs with representative genes of the main functional categories (Figure 7b). Of the induced genes related to amino acid biosynthesis, we selected *STR3* and A*R*G1, which encode the proteins involved in two distinct metabolic pathways, sulfur amino acid and arginine synthesis. We also chose genes related to stress response (*HSP12*), cell wall biosynthesis (*PIR3*), signaling (*BAG7*), and lipid metabolism (*INO1*). As shown in Figure 7b, there was an induction in all these genes in the presence of neomycin, showing in most cases ratios similar to those obtained with DNA microarrays, which validates the transcriptomic results.

**Figure 8:**
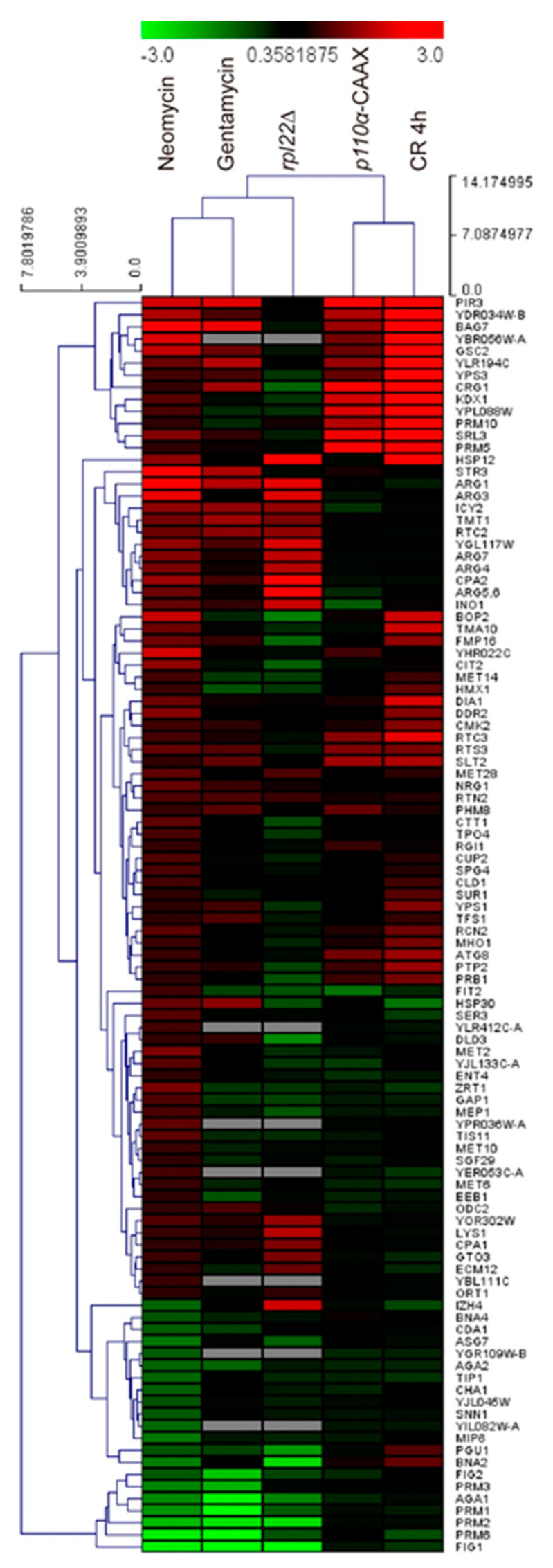
Comparison of the transcriptional profiles in response to neomycin, gentamycin (GEO Accession Number GDS2999), *RPL22* deletion [39], Congo red (CR) exposure [23] and p110α-CAAX overexpression [34]. Red colors are assigned to upregulated genes in each condition while green indicates downregulation. Hierarchical clustering using Euclidean distance was generated using the software Multi-Experiment Viewer (MeV).

We next compared the transcriptional response to neomycin to that observed in cells suffering similar stress conditions to those imposed by neomycin: treatment with the aminoglycoside gentamycin (GEO Accession Number GDS2999), the absence of the ribosomal protein encoding gene *RPL22* [39], overexpression of the plasma membrane-tagged mammalian PI3K-CAAX [34], and cell wall damage due to the chitin-binding dye CR [23]. As observed in Figure 8, treatment with neomycin induced a specific set of genes involved in amino acid metabolism and biosynthesis, as occurred with gentamycin and the *rpl22* mutant, including *ARG1*, *ARG3*, *CPA2*, and/or *LYS1*. These three conditions also shared the repression of genes involved in mating, such as *FIG1*, *AGA1*, or *PRM2*.

Interestingly, neomycin also induced the expression of a group of genes related to cell wall homeostasis and signaling, similar to those induced by CR or triggered by expressing PI3K-CAAX, such as *PIR3, GSC2* (*FKS2*), *BAG7*, and *SLT2*. These are genes that belong to the common transcriptional reprograming fingerprint in response to cell wall stress [40]. Finally, we checked the transcription factors involved in the regulation of the genes differentially expressed in response to neomycin (Tables S6 and S7). As expected, most of them are involved in the modulation of amino acid biosynthetic routes and protein metabolism as well as in the stress response, such as *GCN4* and *YAP1*.

## 3. Discussion

Previously, we showed that rewiring the CWI pathway with the synthetic positive feedback loop IPAC is an excellent tool for identifying novel signaling elements that participate when cells are challenged with an activating stress. Now we have extended the applications of this synthetic circuit to the identification of novel stimuli that fires this pathway. Furthermore, we have optimized the method by integrating the IPAC into the genome, increasing sensitivity by boosting signal amplification.

The hypersensitivity to cell wall stress caused by activation of the IPAC circuit integrated in the yeast genome has allowed us to discover five novel CWI stimuli, namely the aminoglycoside neomycin, lithium chloride (LiCl), zinc chloride (ZnCl_2_), ethylenediaminetetraacetic acid (EDTA), and the antihistamine diphenhydramine (DPH). Neomycin is an antibacterial antibiotic that binds to the 30S subunit of the prokaryotic ribosome inhibiting translation of mRNA, but it also exhibits affinity for phospholipid components of cell membranes, which could lead to alterations at the cell surface [31]. Whereas it is difficult to devise the mechanism of action of the antihistamine DPH as CWI pathway-activating compound, LiCl is known to play a role in two key signal transduction pathways including protein kinase C and glycogen synthase kinase-3 in human cells [41] and to induce competence in yeast cells for DNA transformation [42]. Therefore, it could be stimulating CWI pathway signaling from the yeast cell surface. In the same line, Zn associates with the carbohydrate components of plant cell wall and provokes changes in the cell wall structure [43]. In addition, ZnCl_2_ has been reported to cause oxidative stress in eukaryotic cells by blocking essential enzymatic functional groups [43] and oxidative stress has been shown to stimulate the CWI pathway [4]. ZnCl_2_ could affect not only enzymes involved in stress response but also in cell wall construction, leading to cell wall perturbation. Similarly, EDTA may be inactivating metalloproteins through its ability to chelate divalent metals [44].

Among these compounds, we decided to further investigate the impact of neomycin on yeast cells and determine the role the CWI pathway plays in the cellular response to neomycin, as our results indicated that this antibiotic exerts some specific actions not shared by other protein synthesis inhibitors. Standard grade neomycin, supplied as neomycin sulfate, is a mixture of neomycin A, neomycin B and neomycin C, and some minor compounds found in much smaller amounts. It contains a minimum of 85% neomycin B, which is the most active component, followed by neomycin C and neomycin A, an inactive degradation product of the C and B isomers that does not exhibit antibiotic properties [45]. Although the activity of neomycin C and A in activating the CWI pathway cannot be ruled out, such activity is most likely dependent on the more abundant neomycin B.

We have found that this aminoglycoside appears to interact with anionic phospholipids of the plasma membrane, such as PIP_2_ or PS, probably exerting plasma membrane stress, which would be responsible for the CWI pathway activation triggered by neomycin. Previous in vitro assays have shown that neomycin binds specifically to PIP_2_, causing an increase in membrane fluidity [31,32,46]. In fact, neomycin has been used as a probe to label this phospholipid in membranes [47]. Moreover, neomycin also neutralizes the negative charge of this anionic phosphoinositide, interfering with its physiological interaction with the positively charged PH domain of the mammalian phospholipase C (PLC) and inhibiting PIP_2_ hydrolysis both in vitro and in vivo by a process called electrostatic charge shielding [48]. Our results show that neomycin potentiates the PIP_2_ reduction caused by expression in *S. cerevisiae* of both human PI3K and PLC, which is indicative of its ability to sequester PIP_2_ and compromise the availability of this essential phosphoinositide at the PM, also in yeast cells. The aggregates of PIP_2_ and PS that appear associated to the PM upon neomycin treatment resemble those observed upon a decrease in PM tension caused by the TORC2 inhibitor palmitoylcarnitine. This treatment induces PIP_2_ phase separation into invaginated domains that cause TORC2 inactivation [49]. Indeed, it has been shown that PIP_2_ depletion reduces TORC2 activity [50]. All these observations support the idea that neomycin is interfering with PIP_2_ availability and PM organization, impairing TORC2 activity and consequently TORC2-dependent Ypk1 phosphorylation.

In addition, neomycin can inhibit ion channels in mammalian cells by different mechanisms [51,52,53]. Membrane potential can affect the arrangement of certain lipids on the inner side of the plasma membrane, such as the anionic phospholipids PS or PIP_2_, which directly influence the organization of membrane-anchored signaling elements such as Ras-type GTPases, thus modulating MAPK signaling [54]. Similarly, neomycin could be altering the activity of components involved in CWI pathway signaling located in the yeast plasma membrane via PIP_2_. For example, in *S. cerevisiae* Rho1 and its GEF Rom2 interact with PIP_2_ at the plasma membrane [55,56], so the reduction of this interaction by neomycin could lead to the loss of certain Rho1 functions with repercussions for cell wall integrity, leading to activation of the CWI pathway. In support of this hypothesis, the loss of PIP_2_ upon overexpressing human PI3K promotes the activation of the CWI pathway via Rom2 [34]. Indeed, here we prove that Rom2 also participates in signaling through this pathway in response to neomycin.

As in the case of neomycin, the lack of CWI pathway components, such as Tus1, Rho1, Pkc1, Bck1, Mkk1/2, Slt2, and Swi6, leads to sensitivity to palmitoleic unsaturated fatty acid [57], which suggests that this pathway is involved in the maintenance of PM fluidity homeostasis. Likewise, the alteration of the lipid composition of the plasma membrane due to the lack of inositol and the presence of choline in the medium leads to Slt2 phosphorylation and the Rlm1-mediated transcriptional activation of cell wall genes [58]. Furthermore, in a *slt2Δ* mutant both phosphatidylcholine turnover and synthesis are impaired, and the *pkc1Δ*, *bck1Δ*, and *slt2Δ* mutants show auxotrophy for inositol, indicating again an important role of the CWI pathway in PM lipid homeostasis [58]. Therefore, all evidence points to the activation of the CWI pathway by neomycin being due to the alteration of lipid homeostasis caused by this aminoglycoside and, consequently, the cellular response to this alteration is impaired in the absence of key components of this pathway. This is also supported by our transcriptional data showing a group of genes characteristically induced by the CWI pathway in cell wall-activating conditions, such as Congo red, and upon PIP_2_ depletion imposed by p110α-CAAX.

In addition, the transcriptional profile displayed by yeast cells exposed to neomycin shows that protein synthesis is also impaired to some extent. Indeed, apart from hindering protein synthesis in prokaryotes by irreversibly binding to the 30S ribosomal subunit, specifically to a conserved group of nucleotides of 16S rRNA, reducing translation fidelity [59], aminoglycosides have also been shown to bind to mammalian ribosomes, albeit with lower affinity [60]. In particular, binding to mitochondrial ribosomes, and specifically to 12S rRNA, appears to be one of the main causes of the ototoxicity induced by this type of drug [61,62]. In fact, this reduced specificity is being exploited in the search for new applications of these drugs, including as antiprotozoal or antifungal agents [61].

## 4. Materials and Methods

### 4.1. Strains and Growth Conditions

The *Saccharomyces cerevisiae* strains used in this study are listed in Table S8. Yeast cells were routinely cultured overnight at 180 rpm and 24 or 30 °C in a yeast extract peptone dextrose (YPD) medium (1% yeast extract, 2% peptone, and 2% dextrose) or selective synthetic dextrose (SD) medium (0.17% yeast nitrogen base, 0.5% ammonium sulfate, and 2% dextrose), supplemented appropriately with the required amino acids when the cells carried plasmids. For the expression of *GAL1*-driven genes, synthetic galactose (SG) and synthetic raffinose (SR) media were used, in which glucose was replaced with 2% galactose or 1.5% raffinose, respectively. *GAL1* induction in liquid media was performed by growing cells in SR to mid-exponential phase and then refreshing the cultures to an O.D._600 nm_ of 0.3 directly with SR supplemented with galactose 2% (SR-G) for 5 h.

YPD supplemented with 1M sorbitol was used for culturing CL128 and *pkc1Δ* strains. Overnight stationary phase cultures were refreshed to an O.D._600 nm_ of 0.3 with YPD and incubated for an additional 2–3 h under the same conditions until the exponential growth phase. Then, when necessary, Congo red (Merck, Darmstadt, Germany), neomycin, supplied as neomycin sulfate (Gibco^®^, Thermo Fisher Scientific, Waltham, MA, USA), or other compounds were added at the indicated concentrations, and cells were collected and subsequently processed according to the experimental approach.

### 4.2. DNA Manipulation and Plasmids

To integrate the IPAC cassette [24] into the yeast genome, the plasmid pCWIST-IPAC was constructed in several steps, based on the previously described strategy for integration at the *HO* locus [63]. We used, as a backbone, the pRS306-based custom-made plasmid pCWIST. To obtain pCWIST-IPAC, we first subcloned the *Bam*H1-*Eco*R1 fragment containing the *natmx6* resistance gene from plasmid pFA6a-natMX6 [64] into pCWIST to yield pCWIST-Nat. Then, the IPAC cassette was introduced by successive cloning into pCWIST-Nat the *MLP1* promoter, PCR amplified with oligonucleotides 5′-CCCC*GGGCCC*ACAACAAGAACGTGGGCGATAC-3′ (*Psp*OMI) and 5′-CCCC*CTCGAG*CATTTAATTGTGAATCTTTCTTCG-3′ (*Xho*I), the *MKK1^S386P^* coding sequence, PCR amplified with oligonucleotides 5′-CCCGTCGA*CTCGAG*ATGGCTTCACTGTTCAGACC-3 (*Xho*I) and 5′-CCC*GTCGAC*TTAATCTTTCCAGCACTTCC (*Sal*I) using plasmid pNV7-MKK1^P386^ [22] as template, and the *ADH1* terminator, PCR amplified with oligonucleotides 5′-CCCCGGATCC*GTCGAC*CCTGAGTAATAAGCG-3′ (*Sal*I) and 5′-CCCCC*GGATCC*CGGTGGTGGTCAATAAG-3′ (*Bam*H1). pCWIST-IPAC, after *Not*1 digestion, allowed integration of the cassette (*MLP1*p-*MKK1^S386P^-ADH1*t-*Nat^R^*) at the *HO-SSB1* locus. YSTH1 (Y3656 *HO*::*natMX6*::*SSB1*) and YSTH2 (Y3656 *HO*::*MLP1*p-*MKK1^S386P^-ADH1*t-*natMX6*::*SSB1*) strains were constructed by integrating the *Not*1 fragment from pCWIST-Nat and pCWIST-IPAC, respectively, into the wild-type Y3656 strain.

The plasmids YCplac111 [65], YCplac111-IPAC [24], Yep352-pMLP1-lacZ [25], YCpLG-p110/YCpLG-p110-K802R [37], YCpLG-Myr-p110α [66], and pJMCS-DM23 [36] have already been described. Plasmids pLB187 (empty plasmid) and pLB215 (Ypk1-HA) [10], were kindly provided by Dr. Ted Powers (UC Davis, CA, USA). Plasmid pRS410-GFP-LactC2 was a generous gift from Dr. Sergio Grinstein (University of Toronto, Canada). Plasmids BG1805-PLC1 and pYES2 were acquired from Thermo Scientific Open Biosystems Yeast ORF Collection and Invitrogen (Waltham, MA, USA), respectively.

### 4.3. Halo Sensitivity Assays

A volume of 100 μL of overnight cultures adjusted to O.D._600nm_ = 0.05 was homogeneously distributed on YPD plates, or 50 μL of stationary phase cultures was inoculated into 4 mL of YPD-0.7% agar, and the mixture was poured homogeneously to cover the surface of a YPD plate. Then, sterile 6 mm-diameter filter paper disks previously impregnated with 20 μL of the compound under study were deposited at the appropriate concentration for each experiment. After 48 h of incubation at 30 °C, the diameters of growth inhibition halos generated by diffusion of the compounds from the disk through the agar were measured.

### 4.4. Multiwell Plate Sensitivity Assay

Overnight cultures were diluted to a final O.D._600nm_ of 0.01 within multiwell plates containing YPD with a range of zymolyase 100T (MP Biomedicals) concentrations. Cell suspensions were cultured at 30 °C for 12.5 h in static conditions, and growth was determined as O.D._595nm_ using a Bio-Rad 680 microplate reader (Bio-Rad, Hercules, CA, USA).

### 4.5. Yeast Drop Dilution Growth Assay

Growth assays were performed by spotting onto the surface of YPD, SD, or SG plates 5 μL of 10-fold serial dilutions of overnight cell suspensions adjusted to an initial O.D._600 nm_ of 0.5. Plates were incubated at 30 °C for 48 h.

### 4.6. Preparation of Yeast Extracts and Immunoblotting Analysis

Techniques used for obtaining yeast extracts, fractionation by SDS-PAGE, and transfer to nitrocellulose membranes have been previously described [67].

Immunodetection was carried out with the following primary antibodies: rabbit polyclonal anti-phospho-p44/42 (Thr202/Tyr204) MAPK antibody (Cell Signaling, Danvers, MA, USA) for dually phosphorylated Slt2, mouse monoclonal anti-Slt2 (E-9) antibody (Santa Cruz Biotechnology, Dallas, TX, USA) for total Slt2 protein [67], mouse monoclonal anti-Myc (4A6) antibody (Merck, Darmstadt, Germany,) for Myc-tagged Rlm1, rabbit polyclonal anti-phospho-Ypk1 (T662) (1:20,000), kindly provided by Dr. Ted Powers, for phospho-T662-Ypk1, mouse monoclonal anti-HA 12CA5 (1:1000, Sigma-Aldrich, St. Louis, MO, USA) for HA-tagged Ypk1, and rabbit polyclonal anti-G6PDH antibody (Sigma-Aldrich, He) for G6PDH recognition (loading control). Fluorescent-conjugated secondary antibodies were used to detect primary antibodies with an Odyssey Infrared Imaging System (Li-Cor Biosciences, Lincoln, NE, USA).

### 4.7. β-Galactosidase Activity Assay

Following the procedure described in [25], yeast extracts were obtained by mechanical breakage using glass beads, and the protein concentration was determined by a Bradford assay. β-galactosidase activity was spectrophotometrically measured at 415 nm, using *o*-nitrophenyl-β-D-galactopyranoside as a substrate, and expressed as nmoles of o-nitrophenol·min^−1^·mg^−1^ of total protein.

### 4.8. Fluorescence Microscopy

For the in vivo fluorescence microscopy of cells expressing pJMCS-DM23 (GFP-2XPH-PLCδ1) [36], exponentially growing SR-G cultures treated or not with neomycin 5 mg/mL were collected by centrifugation at 5000 rpm for 1 min and directly observed with an Eclipse TE2000U microscope (Nikon, Tokyo, Japan) using the appropriate sets of filters. For pRS410-GFP-LactC2 observation, the same procedure was followed but cells were cultured in SD. Digital images were acquired with an Orca C4742-95-12ER charge-coupled device camera (Hamamatsu, Hamamatsu city, Japan) and processed with HCImage software (Hamamatsu). Statistics on cell populations were performed by counting >100 cells for each experiment.

### 4.9. Transcriptional Analysis by DNA Microarray Technology

Exponentially growing cells in YPD were treated or not with 10 mg/mL neomycin for 4 h. Total RNA isolation and purification, performed using RNeasy Mini Kit (Qiagen, Hilden, Germany), as well as cDNA synthesis and microarray hybridization using an Affymetrix GeneChip^®^ Yeast Genome 2.0 platform (Affymetrix, Santa Clara, CA, USA), were carried out as previously described [34]. For each experimental condition, three independent biological samples were processed and analyzed.

The fluorescence signal intensities obtained were converted into gene expression values using the GCRMA program (Bioconductor) [68]. To determine the differences in expression levels, a ratio was calculated by dividing the mean of the fluorescence signal of treated cells by one of the untreated control samples. Genes were considered induced or repressed when their expression ratio under the analyzed conditions was ≥1.7 or ≤0.6, respectively. Statistical analyses were performed with Cyber-t software (http://cybert.microarray.ics.uci.edu/; accessed on 21 January 2022) [69], and only those genes with a Bayesian *p*-value ≤ 0.05 were considered significant and analyzed further. The microarray data described here follow the MIAME recommendations (http://www.mged.org; accessed on 21 January 2022) and have been deposited at the National Center for Biotechnology Information gene expression and hybridization array data repository (Gene Expression Omnibus (GEO), http://www.ncbi.nlm.nih.gov/geo/; accessed on 29 July 2022) with the accession number GSE210785.

Induced and repressed genes were grouped into functional categories using the bioinformatic tool GO Slim Term Mapper from the SGD database (www.yeastgenome.org; accessed on 21 January 2022). Clustering of the transcriptional profile obtained under neomycin induction conditions was done by comparing to other similar ones with the MarQ program (MarQ.cnb.csic.es) and the 4.9 version of the MeV MultiExperiment Viewer software (http://www.tm4.org/mev; accessed on 21 January 2022) [70,71]. Transcription factors involved in the gene regulation of the response to neomycin were determined using the Yeastract program (www.yeastract.com; accessed on 21 January 2022).

### 4.10. Reverse Transcription Quantitative Real-Time PCR (RT-qPCR)

RNA isolation was performed with a Total RNA Purification kit (Norgen Biotek, Thorold, ON, Canada) or Macherey–Nagel Nucleospin Mini Kit (Macherey-Nagel, Düren, Germany), and cDNA synthesis was done through reverse transcription of the RNA with the AS Transcription System kit (Promega, Madison, WI, USA).

Real-time qPCR reactions were carried out using the Applied Biosystems 7900HT Fast Real-Time equipment, in 384-multiwell plates with Power SYBR Green PCR Master MIX (Applied Biosystems, Waltham, MA, USA). Primers were donated by Dr. Arroyo’s group (Department of Microbiology and Parasitology, UCM), and sequences are available upon request.

The expression rate for each gene was obtained by normalizing their values with respect to the control gene *ACT1*, and the differential expression ratios were calculated according to the 2^−ΔΔCt^ method described by Livak and Schmittgen, 2001 [72].

## Data Availability

The data presented in this study are openly available in GEO at [http://www.ncbi.nlm.nih.gov/geo/; accessed on 21 January 2022], reference number GSE210785.

## Supplementary Materials

The supporting information can be downloaded at: https://www.mdpi.com/article/10.3390/ijms231911034/s1. References [73,74,75,76] are cited in the supplementary materials.
