# Supplementary material for: Neomycin Interferes with Phosphatidylinositol-4,5-Bisphosphate at the Yeast Plasma Membrane and Activates the Cell Wall Integrity Pathway
Source: Int J Mol Sci. 2022 Sep 20;23(19):11034. doi: 10.3390/ijms231911034 (PMC9569482; doi:10.3390/ijms231911034)
Supplement: Supplementary file 1 [file ijms-23-11034-s001.zip › Figure S1.pdf]

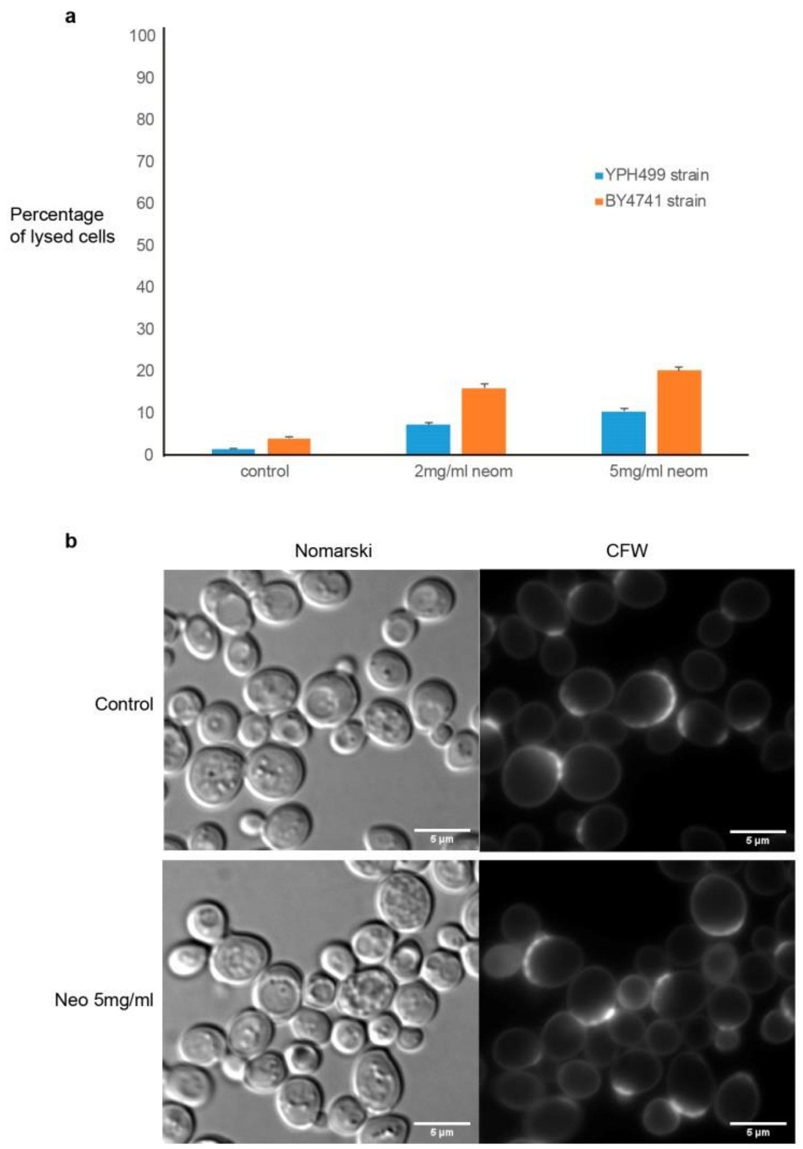

Figure S1. Neomycin slightly increase cell lysis and does not alter the axial budding pattern of yeast cells. (a) Propidium iodide staining of wild type yeast cells (BY4741 and YPH499 strains) cultivated in YPD, treated or not with 2 or 5mg/ml of neomycin for 4 hours and subjected to FACS analysis. At least 10,000 cells were analysed in three biological replicates. (b) Nomarski and fluorescence microscopy images of BY4741 cells treated or not with 5mg/ml of neomycin as in (a), and stained with calcofluor white (CFW).

Materials and Methods for Figure S1.

FACS analysis

Fluorescence Activated Cell Sorting analysis was performed on wild type cells (BY4741 or YPH499) treated or not with 2 or 5 mg/mL for 4 hours and stained with propidium iodide 0.0005% for 5 minutes, washed with PBS twice and diluted 1:10 in PBS previously to the analysis. Cells were analysed in FACScan (Becton Dickinson) with a 670 nm (FL3) emission filter for detecting propidium iodide fluorescence. Data were processed by using the *FlowJo* software.

Fluorescence microscopy of CFW-stained cells

Exponentially growing wild type cells were treated or not with neomycin 5 mg/mL. 1mL of these cultures was incubated with CFW at 5 µg/ml for 10 minutes, washed twice with PBS, collected by centrifugation at 5000 rpm for 1 min and directly observed with an Eclipse TE2000U microscope (Nikon, Tokyo, Japan) using the appropriate set of filters. Digital images were acquired as described in Materials and Methods.
