# Supplementary material for: Neomycin Interferes with Phosphatidylinositol-4,5-Bisphosphate at the Yeast Plasma Membrane and Activates the Cell Wall Integrity Pathway
Source: Int J Mol Sci. 2022 Sep 20;23(19):11034. doi: 10.3390/ijms231911034 (PMC9569482; doi:10.3390/ijms231911034)
Supplement: Supplementary file 1 [file ijms-23-11034-s001.zip › Table S1.pdf]

Table S1. Compounds screened for IPAC activation by halo sensitivity assay.

Compound	Concentration	Solvent
**Ammonium chloride (NH_4Cl)**	481.4 mg/mL	H_2O
**Ammonium persulfate**	22.8 and 228.2 mg/mL	H_2O
**Amphotericin**	10 µg	(disk)
**Atorvastatin**	5 mg/mL	50:50 Methanol:H_2O
**Ascorbic acid**	10 mg/mL	H_2O
**B12 vitamin**	10 mg/mL	H_2O
**Benomil**	150 mg/mL	H_2O
**Benzoyl peroxide**	10 mg/mL	DMSO
**Borax**	10 mg/mL	H_2O
**Boric acid**	10 mg/mL	H_2O
**Butylhydroxy toluene**	10 mg/mL	DMSO
**Caffeine**	20 and 25 mg/mL	H_2O 60 °C
**Calcium chloride ($CaCl_2$)**	111 mg/mL	H_2O
**Calcofluor white**	10 mg/mL	H_2O
**Canavanine**	300 µg/mL	H_2O
**Candesartan**	5 mg/mL	50:50 Methanol:H_2O
**Caspofungin**	20 µg/mL	DMSO
**Cesium chloride (CsCl)**	168.4 mg/mL	H_2O
**Chloramphenicol**	20 mg/mL	Ethanol and acetone
**Chloroquine**	51.6 and 515.9 mg/mL	H_2O
**Cobalt chloride ($CoCl_2$)**	129.8 mg/mL	H_2O
**Congo red**	10 mg/mL	H_2O
**Copper sulphate**	10 mg/mL	H_2O
**Dimethylformamide**	283.2 mg/mL	H_2O
**Diphenhydramine hydrochloride**	25.5 mg/mL	H_2O
**Edetate disodium**	10 mg/mL	H_2O
**EDTA**	8.8 mg/mL	H_2O
**EGTA**	11.4 mg/mL	H_2O
**Erythromycin**	2 mg/mL	H_2O and acetone
**Escitalopram**	5 mg/mL	H_2O

**Ethanol**	394.5 mg/mL	H_2O
**Fluorescein sodium**	10 mg/mL	H_2O
**Formamide**	339.9 mg/mL	H_2O
**Galactose**	200 mg/mL	H_2O
**Geneticin (G418)**	10 mg/mL	H_2O
**Gentamicin sulfate**	10 mg/mL	H_2O
**Glucose**	200 mg/mL	H_2O
**Glycerol**	9.2 and 92.1 mg/mL	H_2O
**Hexaamminecobalt chloride**	26.7 and 267.5 mg/mL	H_2O
**Hydrochloric acid**	0.15 mg/mL	H_2O
**Hydroquinone**	10 mg/mL	H_2O
**Hydroxycortisone base**	10 mg/mL	DMSO
**Hydroxyquinoline**	2 mg/mL	H_2O and ethanol
**Hydroxyurea**	60 mg/mL	H_2O
**Hypoxanthine**	13.6 and 136.1 mg/mL	H_2O
**Kanamycin sulfate**	10 mg/mL	H_2O
**Lidocaine hydrochloride**	10 mg/mL	H_2O
**Lithium acetate**	6.6 and 66 mg/mL	H_2O
**Lithium chloride (LiCl)**	42.4 mg/mL	H_2O
**Lomoxicam**	5 mg/mL	50:50 Methanol:H_2O
**Loratadine**	30 mg/mL	H_2O
**Lysozyme**	20 mg/mL	H_2O
**Magnesium chloride ($MgCl_2$)**	95.2 mg/mL	H_2O
**MES buffer pH 4-9**		H_2O
**H_2O_2**	2.9 mg/mL	H_2O
**Methylparaben**	10 mg/mL	H_2O
**Metronidazole**	10 mg/mL	DMSO
**Minoxidil**	10 mg/mL	DMSO
**Monensin**	67.1 and 670.9 mg/mL	H_2O
**Neomycin sulfate**	10 mg/mL	H_2O
**n-propyl gallate**	21.2 and 212.2 mg/mL	H_2O
**Omeprazole**	5 mg/mL	H_2O
**Orcinol**	12.4 and 124.1 mg/mL	H_2O
**Paracetamol**	10 mg/mL	H_2O
**Phenanthroline**	1 mg/mL	Ethanol and acetone

**Picnic Acid**	10 mg/mL	H_2O
**Ponceau red**	10 mg/mL	H_2O
**Potassium acetate (CH_3COOK)**	98.2 mg/mL	H_2O
**Potassium chloride (KCl)**	969.2 mg/mL	H_2O
**Potassium iodide**	10 mg/mL	H_2O
**Propylparaben**	10 mg/mL	DMSO
**Retinoic acid**	10 mg/mL	H_2O
**Rubidium chloride (RbCl)**	241.8 mg/mL	H_2O
**Salicylic acid**	10 mg/mL	DMSO
**Saponin**	20 mg/mL	H_2O
**SDS**	30 mg/mL	H_2O
**Simvastatin**	5 mg/mL	Polyethylene glycol
**Sodium chloride (NaCl)**	759.7 mg/mL	H_2O
**Sodium fluoride (NaF)**	2.1 mg/mL	H_2O
**Sodium hydrosulfite ($Na_2S_2O_4$)**	10 mg/mL	H_2O
**Sodium orthovanadate**	9.2, 18.4 and 27.6 mg/mL	H_2O
**Sodium sulfite (Na_2SO_3)**	126 mg/mL	H_2O
**Sodium thiosulfate**	15.8 and 158.1 mg/mL	H_2O
**Streptomycin sulfate**	10 mg/mL	H_2O
**Theophylline**	18 and 180.2 mg/mL	H_2O
**Thimerosal**	40.5 and 404.8 mg/mL	H_2O
**Tris-HCl pH 4-10**		H_2O
**Triton**	106 mg/mL	H_2O
**Valsartan**	5 mg/mL	H_2O
**Wortmannina**	1 µg/mL	H_2O
**Xanthohumol**	5 mg/mL	Methanol
**Zinc chloride ($ZnCl_2$)**	136.3 mg/mL	H_2O
**Zinc nitrate**	10 mg/mL	H_2O
**Zymolyase**	50 µg/mL	H_2O
