# Supplementary material for: Neomycin Interferes with Phosphatidylinositol-4,5-Bisphosphate at the Yeast Plasma Membrane and Activates the Cell Wall Integrity Pathway
Source: Int J Mol Sci. 2022 Sep 20;23(19):11034. doi: 10.3390/ijms231911034 (PMC9569482; doi:10.3390/ijms231911034)
Supplement: Supplementary file 1 [file ijms-23-11034-s001.zip › Table S2.pdf]

Table S2. Up-regulated genes differentially expressed upon neomycin treatment of wild type (BY4741) yeast cells.

ORF	Gene	Ratio	Description
**YGL184C**	*STR3*	7.27	Peroxisomal cystathionine beta-lyase, converts cystathionine into homocysteine; may be redox regulated by Gto1p
**YOL058W**	*ARG1*	6.12	Arginosuccinate synthetase, catalyzes the formation of L-argininosuccinate from citrulline and L-aspartate in the arginine biosynthesis pathway; potential Cdc28p substrate
**YOR134W**	*BAG7*	5.20	Rho GTPase activating protein (RhoGAP), stimulates the intrinsic GTPase activity of Rho1p, which plays a role in actin cytoskeleton organization and control of cell wall synthesis; structurally and functionally related to Sac7p
**YJL088W**	*ARG3*	4.49	Ornithine carbamoyltransferase (carbamoylphosphate:L-ornithine carbamoyltransferase), catalyzes the sixth step in the biosynthesis of the arginine precursor ornithine
**YBR056W-A**	---	4.15	---
**YKL163W**	*PIR3*	3.93	O-glycosylated covalently-bound cell wall protein required for cell wall stability; expression is cell cycle regulated, peaking in M/G1 and also subject to regulation by the cell integrity pathway
**YHR022C**	---	3.88	---
**YLR267W**	*BOP2*	3.65	Protein of unknown function
**YGR032W**	*GSC2*	3.59	Catalytic subunit of 1,3-beta-glucan synthase, involved in formation of the inner layer of the spore wall; activity positively regulated by Rho1p and negatively by Smk1p; has similarity to an alternate catalytic subunit, Fks1p (Gsc1p)
**YDR034W-B**	---	3.25	---
**YJR109C**	*CPA2*	2.91	Large subunit of carbamoyl phosphate synthetase, which catalyzes a step in the synthesis of citrulline, an arginine precursor
**YGL117W**	---	2.74	---
**YPL250C**	*ICY2*	2.74	Protein of unknown function; mobilized into polysomes upon a shift from a fermentable to nonfermentable carbon source; potential Cdc28p substrate
**YCR005C**	*CIT2*	2.73	Citrate synthase, catalyzes the condensation of acetyl coenzyme A and oxaloacetate to form citrate, peroxisomal isozyme involved in glyoxylate cycle; expression is controlled by Rtg1p and Rtg2p transcription factors
**YFL014W**	*HSP12*	2.72	Plasma membrane protein involved in maintaining membrane organization in stress conditions; induced by heat shock, oxidative stress, osmostress, stationary phase, glucose depletion, oleate and alcohol; regulated by HOG and Ras-Pka pathways
**YOL052C-A**	*DDR2*	2.61	Multistress response protein, expression is activated by a variety of xenobiotic agents and environmental or physiological stresses
**YMR062C**	*ARG7*	2.60	Mitochondrial ornithine acetyltransferase, catalyzes the fifth step in arginine biosynthesis; also possesses acetylglutamate synthase activity, regenerates acetylglutamate while forming ornithine
**YKR091W**	*SRL3*	2.56	Cytoplasmic protein that, when overexpressed, suppresses the lethality of a rad53 null mutation; potential Cdc28p substrate
**YNL277W**	*MET2*	2.49	L-homoserine-O-acetyltransferase, catalyzes the conversion of homoserine to O-acetyl homoserine which is the first step of the methionine biosynthetic pathway
**YHR018C**	*ARG4*	2.48	Argininosuccinate lyase, catalyzes the final step in the arginine biosynthesis pathway

**YGL255W**	*ZRT1*	2.44	High-affinity zinc transporter of the plasma membrane, responsible for the majority of zinc uptake; transcription is induced under low-zinc conditions by the Zap1p transcription factor
**YER175C**	*TMT1*	2.43	Trans-aconitate methyltransferase, cytosolic enzyme that catalyzes the methyl esterification of 3-isopropylmalate, an intermediate of the leucine biosynthetic pathway, and trans-aconitate, which inhibits the citric acid cycle
**YLR327C**	*TMA10*	2.32	Protein of unknown function that associates with ribosomes; putative homolog of the F1F0-ATPase synthase regulator Stf2p
**YDR070C**	*FMP16*	2.31	Putative protein of unknown function; proposed to be involved in responding to conditions of stress; the authentic, non-tagged protein is detected in highly purified mitochondria in high-throughput studies
**YMR316W**	*DIA1*	2.30	Protein of unknown function, involved in invasive and pseudohyphal growth; green fluorescent protein (GFP)-fusion protein localizes to the cytoplasm in a punctate pattern
**YBR147W**	*RTC2*	2.28	Protein of unknown function; mutant produces large lipid droplets, is resistant to fluconazole, has decreased levels of Rdna transcription, growth defects on minimal media, and suppresses cdc13-1; detected in highly purified mitochondria; similar to a G-protein coupled receptor from S. pombe
**YER069W**	*ARG56*	2.26	Acetylglutamate kinase and N-acetyl-gamma-glutamyl-phosphate reductase; N-acetyl-L-glutamate kinase (NAGK) catalyzes the 2nd and N-acetyl-gamma-glutamyl-phosphate reductase (NAGSA), the 3rd step in arginine biosynthesis; synthesized as a precursor which is processed in the mitochondrion to yield mature NAGK and NAGSA; enzymes form a metabolon complex with Arg2p; NAGK C-terminal domain stabilizes the enzymes, slows catalysis and is involved in feed-back inhibition by arginine
**YLR194C**	---	2.25	---
**YCR021C**	*HSP30*	2.23	Hydrophobic plasma membrane localized, stress-responsive protein that negatively regulates the H(+)-ATPase Pma1p; induced by heat shock, ethanol treatment, weak organic acid, glucose limitation, and entry into stationary phase
**YGR161C**	*RTS3*	2.23	Putative component of the protein phosphatase type 2A complex
**YJL153C**	*INO1*	2.21	Inositol-3-phosphate synthase, involved in synthesis of inositol phosphates and inositol-containing phospholipids; transcription is coregulated with other phospholipid biosynthetic genes by Ino2p and Ino4p, which bind the UASINO DNA element
**YDR043C**	*NRG1*	2.20	Transcriptional repressor that recruits the Cyc8p-Tup1p complex to promoters; mediates glucose repression and negatively regulates a variety of processes including filamentous growth and alkaline Ph response
**YPR036W-A**	---	2.18	---
**YGR088W**	*CTT1*	2.14	Cytosolic catalase T, has a role in protection from oxidative damage by hydrogen peroxide
**YIR017C**	*MET28*	2.12	Basic leucine zipper (Bzip) transcriptional activator in the Cbf1p-Met4p-Met28p complex, participates in the regulation of sulfur metabolism
**YLR136C**	*TIS11*	2.12	Mrna-binding protein expressed during iron starvation; binds to a sequence element in the 3'-untranslated regions of specific mRNAs to mediate their degradation; involved in iron homeostasis
**YOR220W**	*RCN2*	2.11	Protein of unknown function; green fluorescent protein (GFP)-fusion protein localizes to the cytoplasm and is induced in response to the DNA-damaging agent MMS; phosphorylated in response to alpha factor
**YJL133C-A**	---	2.09	---
**YKL161C**	*KDX1*	2.08	Protein kinase implicated in the Slt2p mitogen-activated (MAP) kinase signaling pathway; interacts with numerous components in the mating pheromone and CWI MAPK pathways; associates with Rlm1p

**YER081W**	*SER3*	2.05	3-phosphoglycerate dehydrogenase, catalyzes the first step in serine and glycine biosynthesis; isozyme of Ser33p
**YOR302W**	---	2.02	---
**YKR039W**	*GAP1*	2.01	General amino acid permease; Gap1p senses the presence of amino acid substrates to regulate localization to the plasma membrane when needed
**YDL204W**	*RTN2*	1.98	Reticulon protein that stabilizes membrane curvature; interacts with Rtn1p, Yop1p, and Sey1p to maintain tubular ER morphology; involved in nuclear pore assembly; rtn1 rtn2 yop1 triple mutant shows loss of tubular ER; interacts with exocyst subunit Sec6p, with Yip3p, and with Sbh1p; similar to and much less abundant than Rtn1p; member of the RTNLA (reticulon-like A) subfamily
**YGL166W**	*CUP2*	1.98	Copper-binding transcription factor; activates transcription of the metallothionein genes CUP1-1 and CUP1-2 in response to elevated copper concentrations
**YPL088W**	---	1.96	---
**YGR121C**	*MEP1*	1.96	Ammonium permease; belongs to a ubiquitous family of cytoplasmic membrane proteins that transport only ammonium (NH4+); expression is under the nitrogen catabolite repression regulation
**YMR107W**	*SPG4*	1.94	Protein required for survival at high temperature during stationary phase; not required for growth on nonfermentable carbon sources
**YOR273C**	*TPO4*	1.93	Polyamine transport protein, recognizes spermine, putrescine, and spermidine; localizes to the plasma membrane; member of the major facilitator superfamily
**YHR087W**	*RTC3*	1.90	Protein of unknown function involved in RNA metabolism; has structural similarity to SBDS, the human protein mutated in Shwachman-Diamond Syndrome (the yeast SBDS ortholog = SDO1); null mutation suppresses cdc13-1 temperature sensitivity
**YER053C-A**	---	1.90	---
**YGR110W**	*CLD1*	1.89	Mitochondrial cardiolipin-specific phospholipase; functions upstream of Taz1p to generate monolyso-cardiolipin; transcription increases upon genotoxic stress; involved in restricting Ty1 transposition; has homology to mammalian CGI-58
**YLR121C**	*YPS3*	1.89	Aspartic protease, member of the yapsin family of proteases involved in cell wall growth and maintenance; attached to the plasma membrane via a glycosylphosphatidylinositol (GPI) anchor
**YKL001C**	*MET14*	1.88	Adenylylsulfate kinase, required for sulfate assimilation and involved in methionine metabolism
**YLR412C-A**	---	1.88	---
**YOL016C**	*CMK2*	1.88	Calmodulin-dependent protein kinase; may play a role in stress response, many CA++/3almodulin dependent phosphorylation substrates demonstrated in vitro, amino acid sequence similar to Cmk1p and mammalian Cam Kinase II
**YOR382W**	*FIT2*	1.87	Mannoprotein that is incorporated into the cell wall via a glycosylphosphatidylinositol (GPI) anchor, involved in the retention of siderophore-iron in the cell wall
**YJR008W**	*MHO1*	1.84	Protein of unknown function; inhibits haploid invasive growth when overexpressed; synthetically lethal with phospholipase C (PLC1); expression induced by mild heat-stress on a non-fermentable carbon source, upon entry into stationary phase and upon nitrogen deprivation; repressed by inosine and choline in an Opi1p-dependent manner; highly conserved from bacteria to human; Memo, the human homolog, is an ErbB2 interacting protein with an essential function in cell motility

**YFR030W**	*MET10*	1.83	Subunit alpha of assimilatory sulfite reductase, which converts sulfite into sulfide
**YBL111C**		1.83	Helicase-like protein encoded within the telomeric Y' element; relocalizes from mitochondrion to cytoplasm upon DNA replication stress.
**YOR208W**	*PTP2*	1.83	Phosphotyrosine-specific protein phosphatase involved in the inactivation of mitogen-activated protein kinase (MAPK) during osmolarity sensing; dephosporylates Hog1p MAPK and regulates its localization; localized to the nucleus
**YMR251W**	*GTO3*	1.82	Omega class glutathione transferase; putative cytosolic localization
**YBL078C**	*ATG8*	1.81	Component of autophagosomes and Cvt vesicles; undergoes conjugation to phosphatidylethanolamine (PE); Atg8p-PE is anchored to membranes, is involved in phagophore expansion, and may mediate membrane fusion during autophagosome formation
**YLR205C**	*HMX1*	1.81	ER localized heme oxygenase, involved in heme degradation during iron starvation and in the oxidative stress response; expression is regulated by AFT1 and oxidative stress; relocates to the perinuclear region in the presence of oxidants
**YER091C**	*MET6*	1.80	Cobalamin-independent methionine synthase, involved in methionine biosynthesis and regeneration; requires a minimum of two glutamates on the methyltetrahydrofolate substrate, similar to bacterial metE homologs
**YIR034C**	*LYS1*	1.80	Saccharopine dehydrogenase (NAD+, L-lysine-forming), catalyzes the conversion of saccharopine to L-lysine, which is the final step in the lysine biosynthesis pathway; also has Mrna binding activity
**YER037W**	*PHM8*	1.79	Lysophosphatidic acid (LPA) phosphatase involved in LPA hydrolysis in response to phosphate starvation; phosphatase activity is soluble and Mg2+ dependent; expression is induced by low phosphate levels and by inactivation of Pho85p
**YER067W**	*RGI1*	1.78	Protein of unknown function involved in energy metabolism under respiratory conditions; protein abundance is increased upon intracellular iron depletion
**YHR209W**	*CRG1*	1.78	S-AdoMet-dependent methyltransferase involved in lipid homeostasis; mediates resistance to a drug cantharidin
**YHR030C**	*SLT2*	1.77	Serine/threonine MAP kinase; involved in regulating maintenance of cell wall integrity, progression through the cell cycle, and nuclear Mrna retention in heat shock; required for mitophagy and pexophagy; affects recruitment of mitochondria to the phagophore assembly site (PAS); regulated by the PKC1-mediated signaling pathway
**YOR222W**	*ODC2*	1.76	Mitochondrial inner membrane transporter, exports 2-oxoadipate and 2-oxoglutarate from the mitochondrial matrix to the cytosol for use in lysine and glutamate biosynthesis and in lysine catabolism
**YHR021W-A**	*ECM12*	1.76	Putative protein of unknown function; may contribute to cell wall biosynthesis, mutants display zymolyase hypersensitivity
**YCL010C**	*SGF29*	1.75	Component of the HAT/Core module of the SAGA, SLIK, and ADA complexes; HAT/Core module also contains Gcn5p, Ngg1p, and Ada2p; binds methylated histone H3K4; involved in transcriptional regulation through SAGA and TBP recruitment to target promoters and H3 acetylation
**YOR303W**	*CPA1*	1.75	Small subunit of carbamoyl phosphate synthetase, which catalyzes a step in the synthesis of citrulline, an arginine precursor; translationally regulated by an attenuator peptide encoded by YOR302W within the CPA1 Mrna 5'-leader

**YEL071W**	*DLD3*	1.75	D-lactate dehydrogenase, part of the retrograde regulon which consists of genes whose expression is stimulated by damage to mitochondria and reduced in cells grown with glutamate as the sole nitrogen source, located in the cytoplasm
**YJL108C**	*PRM10*	1.74	Pheromone-regulated protein, proposed to be involved in mating; predicted to have 5 transmembrane segments; induced by treatment with 8-methoxypsoralen and UVA irradiation
**YLL038C**	*ENT4*	1.74	Protein of unknown function, contains an N-terminal epsin-like domain; proposed to be involved in the trafficking of Arn1p in the absence of ferrichrome
**YIL117C**	*PRM5*	1.73	Pheromone-regulated protein, predicted to have 1 transmembrane segment; induced during cell integrity signaling
**YLR120C**	*YPS1*	1.73	Aspartic protease, member of the yapsin family of proteases involved in cell wall growth and maintenance; attached to the plasma membrane via a glycosylphosphatidylinositol (GPI) anchor
**YLR178C**	*TFS1*	1.72	Protein that interacts with and inhibits carboxypeptidase Y and Ira2p; phosphatidylethanolamine-binding protein (PEBP) family member; targets to vacuolar membranes during stationary phase; acetylated by NatB N-terminal acetyltransferase
**YPL057C**	*SUR1*	1.72	Probable catalytic subunit of a mannosylinositol phosphorylceramide (MIPC) synthase, forms a complex with probable regulatory subunit Csg2p; function in sphingolipid biosynthesis is overlapping with that of Csh1p
**YPL095C**	*EEB1*	1.72	Acyl-coenzymeA:ethanol O-acyltransferase responsible for the major part of medium-chain fatty acid ethyl ester biosynthesis during fermentation; possesses short-chain esterase activity; may be involved in lipid metabolism and detoxification
**YEL060C**	*PRB1*	1.72	Vacuolar proteinase B (yscB), a serine protease of the subtilisin family; involved in protein degradation in the vacuole and required for full protein degradation during sporulation; activity inhibited by Pbi2p
**YOR130C**	*ORT1*	1.72	Ornithine transporter of the mitochondrial inner membrane, exports ornithine from mitochondria as part of arginine biosynthesis; human ortholog is associated with hyperammonaemia-hyperornithinaemia-homocitrullinuria (HHH) syndrome
