# Supplementary material for: Neomycin Interferes with Phosphatidylinositol-4,5-Bisphosphate at the Yeast Plasma Membrane and Activates the Cell Wall Integrity Pathway
Source: Int J Mol Sci. 2022 Sep 20;23(19):11034. doi: 10.3390/ijms231911034 (PMC9569482; doi:10.3390/ijms231911034)
Supplement: Supplementary file 1 [file ijms-23-11034-s001.zip › Table S3.pdf]

Table S3. Down-regulated genes differentially expressed upon neomycin treatment of wild type (BY4741) yeast cells.

ORF	Gene	Ratio	Description
**YBL098W**	*BNA4*	0.59	Kynurenine 3-mono oxygenase, required for the de novo biosynthesis of NAD from tryptophan via kynurenine; expression regulated by Hst1p; putative therapeutic target for Huntington disease
**YJL045W**	---	0.59	---
**YJR153W**	*PGU1*	0.58	Endo-polygalacturonase, pectolytic enzyme that hydrolyzes the alpha-1,4-glycosidic bonds in the rhamnogalacturonan chains in pectins
**YCR089W**	*FIG2*	0.57	Cell wall adhesin, expressed specifically during mating; may be involved in maintenance of cell wall integrity during mating
**YNL086W**	*SNN1*	0.56	Putative protein of unknown function; likely member of BLOC complex involved in endosomal cargo sorting; green fluorescent protein (GFP)-fusion protein localizes to endosomes
**YGR109W-B**	---	0.56	---
**YCL064C**	*CHA1*	0.54	Catabolic L-serine (L-threonine) deaminase, catalyzes the degradation of both L-serine and L-threonine; required to use serine or threonine as the sole nitrogen source, transcriptionally induced by serine and threonine
**YLR307W**	*CDA1*	0.54	Chitin deacetylase, together with Cda2p involved in the biosynthesis ascospore wall component, chitosan; required for proper rigidity of the ascospore wall
**YOL101C**	*IZH4*	0.52	Membrane protein involved in zinc ion homeostasis, member of the four-protein IZH family, expression induced by fatty acids and altered zinc levels; deletion reduces sensitivity to excess zinc; possible role in sterol metabolism
**YGL032C**	*AGA2*	0.52	Adhesion subunit of a-agglutinin of a-cells, C-terminal sequence acts as a ligand for alpha-agglutinin (Sag1p) during agglutination, modified with O-linked oligomannosyl chains, linked to anchorage subunit Aga1p via two disulfide bonds
**YBR067C**	*TIP1*	0.51	Major cell wall mannoprotein with possible lipase activity; transcription is induced by heat- and cold-shock; member of the Srp1p/Tip1p family of serine-alanine-rich proteins
**YIL082W-A**	---	0.49	---
**YJL170C**	*ASG7*	0.45	Protein that regulates signaling from a G protein beta subunit Ste4p and its relocalization within the cell; specific to a-cells and induced by alpha-factor
**YHR015W**	*MIP6*	0.44	Putative RNA-binding protein, interacts with Mex67p, which is a component of the nuclear pore involved in nuclear mRNA export
**YJR078W**	*BNA2*	0.41	Putative tryptophan 2,3-dioxygenase or indoleamine 2,3-dioxygenase, required for de novo biosynthesis of NAD from tryptophan via kynurenine; interacts genetically with telomere capping gene CDC13; regulated by Hst1p and Aftp
**YPL192C**	*PRM3*	0.39	Pheromone-regulated protein required for nuclear envelope fusion during karyogamy; localizes to the outer face of the nuclear membrane; interacts with Kar5p at the spindle pole body

**YNR044W**	*AGA1*	0.37	Anchorage subunit of a-agglutinin of a-cells, highly O-glycosylated protein with N-terminal secretion signal and C-terminal signal for addition of GPI anchor to cell wall, linked to adhesion subunit Aga2p via two disulfide bonds
**YNL279W**	*PRM1*	0.34	Pheromone-regulated multispanning membrane protein involved in membrane fusion during mating; predicted to have 5 transmembrane segments and a coiled coil domain; localizes to the shmoo tip; regulated by Ste12p
**YIL037C**	*PRM2*	0.24	Pheromone-regulated protein, predicted to have 4 transmembrane segments and a coiled coil domain; regulated by Ste12p; required for efficient nuclear fusion
**YML047C**	*PRM6*	0.15	Pheromone-regulated protein, predicted to have 2 transmembrane segments; regulated by Ste12p during mating
**YBR040W**	*FIG1*	0.14	Integral membrane protein required for efficient mating; may participate in or regulate the low affinity Ca2+ influx system, which affects intracellular signaling and cell-cell fusion during mating
