# Supplementary material for: Neomycin Interferes with Phosphatidylinositol-4,5-Bisphosphate at the Yeast Plasma Membrane and Activates the Cell Wall Integrity Pathway
Source: Int J Mol Sci. 2022 Sep 20;23(19):11034. doi: 10.3390/ijms231911034 (PMC9569482; doi:10.3390/ijms231911034)
Supplement: Supplementary file 1 [file ijms-23-11034-s001.zip › Table S4.pdf]

Table S4. Gene Ontology term enrichment within the up-regulated genes in response to neomycin exposure. For each of the Gene Ontology (GO) term with its corresponding reference, is indicated the percentage of genes related to this GO term within the genes up-regulated, the percentage of genes with this GO annotation within the overall *S. cerevisiae* genome and the names of the genes annotated to this term in our list of neomycin-induced genes. Only GO terms that show an enrichment statistically significant according to a $\chi 2$ test (*p*-values ≤ 0.05) are shown. The analysis was performed by using the tool GO Slim mapper from the SGD database.

GO term	% in cluster	% in *S. cerevisiae* genome	Genes annotated to the term
**Cellular amino acid metabolic process (GO:0006520)**	13 of 83 genes, 15.66%	155 of 6486 annotated genes, 2.39%	*ARG1, ARG3, ARG4, ARG56, ARG7, CPA1, CPA2, LYS1, MET2, MET6, ORT1, SER3, STR3*
**Membrane invagination (GO: 0010324)**	1 of 83 genes, 1.20%	1 of 6486 annotated genes, 0.02%	*ATG8*
**Response to starvation (GO:0042594)**	4 of 83 genes, 4.82%	61 of 6486 annotated genes, 0.94%	*DDR2, PHM8, PRB1, TMT1*
**Amino acid transport (GO:0006865)**	3 of 83 genes, 3.61%	44 of 6486 annotated genes, 0.68%	*GAP1, ORT1, RTC2*
