# Supplementary material for: Neomycin Interferes with Phosphatidylinositol-4,5-Bisphosphate at the Yeast Plasma Membrane and Activates the Cell Wall Integrity Pathway
Source: Int J Mol Sci. 2022 Sep 20;23(19):11034. doi: 10.3390/ijms231911034 (PMC9569482; doi:10.3390/ijms231911034)
Supplement: Supplementary file 1 [file ijms-23-11034-s001.zip › Table S5.pdf]

Table S5. Gene Ontology term enrichment within the genes down-regulated in response to neomycin exposure. For each of the Gene Ontology (GO) term with its corresponding reference, is indicated the percentage of genes related to this GO term within the down-regulated genes, the percentage of genes with this GO annotation within the overall *S. cerevisiae* genome and the names of the genes annotated to this term in the list of neomycin down-regulated genes. Only GO terms that show an enrichment statistically significant according to a $\chi 2$ test (p-values ≤ 0.05) are shown. The analysis was performed by using the tool GO Slim mapper from the SGD database.

Gene Ontology (GO) term	% in cluster	% in *S. cerevisiae* genome	Genes annotated to the term
**conjugation (GO: 0000746)**	8 of 21 genes, 38.10%	121 of 6486 annotated genes, 1.87%	*AGA1, AGA2, ASG7, FIG1, FIG2, PRM1, PRM2, PRM3*
**cell morphogenesis (GO:0000902)**	2 of 21 genes, 9.52%	28 of 6486 annotated genes, 0.43%	*FIG1, FIG2*
**membrane fusion (GO:0061025)**	2 of 21 genes, 9.52%	51 of 6486 annotated genes, 0.79%	*PRM1, PRM3*
**cellular amino acid metabolic process (GO:0006520)**	3 of 21 genes, 14.29%	155 of 6486 annotated genes, 2.39%	*BNA2, BNA4, CHA1*
**nucleus organization (GO:0006997)**	2 of 21 genes, 9.52%	96 of 6486 annotated genes, 1.48%	*PRM2, PRM3*
**organelle fusion (GO:0048284)**	2 of 21 genes, 9.52%	99 of 6486 annotated genes, 1.53%	*PRM2, PRM3*
