# Supplementary material for: Neomycin Interferes with Phosphatidylinositol-4,5-Bisphosphate at the Yeast Plasma Membrane and Activates the Cell Wall Integrity Pathway
Source: Int J Mol Sci. 2022 Sep 20;23(19):11034. doi: 10.3390/ijms231911034 (PMC9569482; doi:10.3390/ijms231911034)
Supplement: Supplementary file 1 [file ijms-23-11034-s001.zip › Table S6.pdf]

Table S6. Transcription factors involved in the regulation of the up-regulated genes in response to neomycin. The table indicates for each transcription factor its function, the percentage of genes regulated in the list of neomycin-induced genes, the percentage regulated in the overall *S. cerevisiae* genome and the name of the genes regulated by them. Only those transcription factors that show an statistically significant enrichment according to a $\chi 2$ test (p-values ≤ 0.05) are shown. The analysis was performed by using the tool Search for Transcription Factors at Yeastract website.

Transcription Factor	Function	% in cluster	% in *S. cerevisiae* genome	Genes regulated
**Gcn4**	Basic leucine zipper (bZIP) transcriptional activator of amino acid biosynthetic genes in response to amino acid starvation; expression is tightly regulated at both the transcriptional and translational levels	97,56%	1,32%	*RTC2 SGF29 CIT2 HSP30 RTN2 YDR034W-B NRG1 FMP16 DLD3 PRB1 PHM8 RGI1 SER3 MET6 TMT1 HSP12 MET10 ZRT1 STR3 CUP2 YGL117W GSC2 CTT1 CLD1 MEP1 RTS3 ARG4 ECM12 YHR022C SLT2 RTC3 CRG1 PRM5 MET28 LYS1 INO1 PRM10 MHO1 CPA2 PIR3 KDX1 MET14 GAP1 SRL3 ENT4 YPS1 YPS3 TIS11 TFS1 NCW2 HMX1 BOP2 TMA10 ARG7 SPG4 GTO3 DIA1 MET2 ARG1 DDR2 CMK2 ORT1 BAG7 PTP2 RCN2 ODC2 TPO4 YOR302W CPA1 FIT2 ATG41 EEB1 YPL088W SUR1 YBL111C ATG8 ARG3 YER053C-A SPO24 MNC1*
**Yap1**	Basic leucine zipper (bZIP) transcription factor required for oxidative stress tolerance; activated by H2O2 through the multistep formation of disulfide bonds and transit from the cytoplasm to the nucleus; mediates resistance to cadmium	97,56%	1,33%	*RTC2 SGF29 CIT2 HSP30 RTN2 YDR034W-B NRG1 FMP16 DLD3 PRB1 PHM8 RGI1 SER3 MET6 TMT1 HSP12 MET10 ZRT1 STR3 CUP2 YGL117W GSC2 CTT1 CLD1 MEP1 RTS3 ARG4 ECM12 YHR022C SLT2 RTC3 CRG1 PRM5 MET28 LYS1 INO1 PRM10 MHO1 CPA2 PIR3 KDX1 MET14 GAP1 SRL3 ENT4 YPS1 YPS3 TIS11 TFS1 NCW2 HMX1 BOP2 TMA10 ARG7 SPG4 GTO3 DIA1 MET2 ARG1 DDR2 CMK2 ORT1 BAG7 PTP2 RCN2 ODC2 TPO4 YOR302W CPA1 FIT2 ATG41 EEB1 YPL088W SUR1 YBL111C ATG8 ARG3 YER053C-A SPO24 YLR412C-A*
**Ste12**	Transcription factor that is activated by a MAP kinase signaling cascade, activates genes involved in mating or pseudohyphal/invasive growth pathways; cooperates with Tec1p transcription factor to regulate genes specific for invasive growth	74,39%	1,59%	*RTC2 CIT2 HSP30 RTN2 YDR034W-B NRG1 FMP16 PRB1 PHM8 RGI1 SER3 MET6 HSP12 STR3 CUP2 YGL117W GSC2 CTT1 CLD1 MEP1 RTS3 ARG4 YHR022C SLT2 RTC3 CRG1 PRM5 MET28 INO1 PRM10 PIR3 KDX1 GAP1 SRL3 ENT4 YPS1 YPS3 TIS11 NCW2 HMX1 TMA10 ARG7 SPG4 GTO3 MET2 ARG1 DDR2 ORT1 RCN2 ODC2 TPO4 ATG41 EEB1 YPL088W SUR1 YBL111C ARG3 YER053C-A DPI8 MNC1 YLR412C-A*
**Tec1**	Transcription factor required for full Ty1 expression, Ty1-mediated gene activation, and haploid invasive and diploid pseudohyphal growth; TEA/ATTS DNA-binding domain family member	75,61%	1,62%	*RTC2 CIT2 HSP30 RTN2 NRG1 FMP16 PHM8 RGI1 SER3 TMT1 HSP12 MET10 ZRT1 STR3 CUP2 GSC2 CTT1 CLD1 MEP1 RTS3 YHR022C SLT2 RTC3 CRG1 PRM5 MET28 INO1 PRM10 CPA2 PIR3 KDX1 MET14 GAP1 SRL3 YPS1 YPS3 TIS11 TFS1 NCW2 TMA10 ARG7 SPG4 GTO3 DIA1 MET2 ARG1 DDR2 CMK2 BAG7 PTP2 RCN2 ODC2 TPO4 FIT2 ATG41 EEB1 YPL088W SUR1 ARG3 YER053C-A SPO24 YLR412C-A*

**Sfp1**	Transcription factor that controls expression of ribosome biogenesis genes in response to nutrients and stress, regulates G2/M transitions during mitotic cell cycle and DNA-damage response, modulates cell size; regulated by TORC1 and Mrs6p; can form the [ISP+] prion	74,39%	1,86%	*RTC2 CIT2 HSP30 RTN2 YDR034W-B NRG1 FMP16 DLD3 PRB1 PHM8 SER3 MET6 TMT1 HSP12 MET10 ZRT1 STR3 YGL117W GSC2 CTT1 CLD1 MEP1 RTS3 ARG4 ECM12 YHR022C RTC3 CRG1 PRM5 LYS1 INO1 PRM10 MHO1 CPA2 PIR3 KDX1 MET14 GAP1 YPS1 YPS3 TIS11 TFS1 NCW2 HMX1 TMA10 ARG7 SPG4 DIA1 ARG1 ORT1 ODC2 TPO4 YOR302W FIT2 ATG41 EEB1 YPL088W YBL111C ATG8 ARG3 MNC1*
**Msn2**	Transcriptional activator related to Msn4p; activated in stress conditions, which results in translocation from the cytoplasm to the nucleus; binds DNA at stress response elements of responsive genes, inducing gene expression	89,02%	2,24%	*RTC2 CIT2 HSP30 RTN2 YDR034W-B NRG1 FMP16 DLD3 PRB1 PHM8 RGI1 SER3 MET6 HSP12 MET10 ZRT1 STR3 CUP2 YGL117W GSC2 CTT1 CLD1 MEP1 RTS3 ARG4 ECM12 SLT2 RTC3 CRG1 PRM5 MET28 LYS1 INO1 PRM10 MHO1 CPA2 PIR3 KDX1 MET14 GAP1 YPS1 YPS3 TIS11 TFS1 NCW2 HMX1 BOP2 TMA10 ARG7 SPG4 GTO3 DIA1 MET2 ARG1 DDR2 ORT1 BAG7 RCN2 ODC2 TPO4 YOR302W CPA1 FIT2 ATG41 EEB1 YPL088W SUR1 YBL111C ATG8 ARG3 YER053C-A DPI8 SPO24*
**Bas1**	Myb-related transcription factor involved in regulating basal and induced expression of genes of the purine and histidine biosynthesis pathways; also involved in regulation of meiotic recombination at specific genes	84,15%	2,30%	*RTC2 CIT2 RTN2 NRG1 FMP16 DLD3 PRB1 PHM8 SER3 MET6 TMT1 HSP12 ZRT1 STR3 CUP2 YGL117W GSC2 CTT1 CLD1 MEP1 RTS3 ECM12 YHR022C SLT2 RTC3 CRG1 PRM5 MET28 LYS1 INO1 PRM10 MHO1 CPA2 PIR3 KDX1 GAP1 SRL3 YPS3 TIS11 TFS1 NCW2 HMX1 BOP2 TMA10 ARG7 SPG4 GTO3 DIA1 MET2 ARG1 DDR2 CMK2 ORT1 BAG7 PTP2 RCN2 ODC2 TPO4 YOR302W CPA1 FIT2 ATG41 EEB1 YPL088W ATG8 ARG3 YER053C-A DPI8 SPO24*
**Rap1**	DNA-binding protein involved in either activation or repression of transcription, depending on binding site context; also binds telomere sequences and plays a role in telomeric position effect (silencing) and telomere structure	69,51%	2,06%	*RTC2 CIT2 HSP30 RTN2 NRG1 FMP16 DLD3 PHM8 SER3 MET6 HSP12 ZRT1 STR3 CUP2 GSC2 CTT1 CLD1 RTS3 ECM12 CRG1 PRM5 MET28 LYS1 INO1 MHO1 CPA2 KDX1 MET14 ENT4 TIS11 TFS1 NCW2 BOP2 TMA10 SPG4 GTO3 DIA1 MET2 ARG1 DDR2 CMK2 PTP2 RCN2 TPO4 YOR302W CPA1 FIT2 ATG41 EEB1 YPL088W SUR1 YBL111C ARG3 YER053C-A DPI8 SPO24 YLR412C-A*
**Fkh1**	Forkhead family transcription factor with a minor role in the expression of G2/M phase genes; negatively regulates transcriptional elongation; positive role in chromatin silencing at HML and HMR; regulates donor preference during switching	60,98%	1,89%	*RTC2 SGF29 CIT2 HSP30 RTN2 YDR034W-B DLD3 PRB1 PHM8 RGI1 SER3 MET6 TMT1 HSP12 MET10 ZRT1 STR3 YGL117W CTT1 MEP1 RTS3 ARG4 RTC3 CRG1 MET28 LYS1 INO1 MHO1 CPA2 MET14 GAP1 TFS1 NCW2 HMX1 TMA10 ARG7 SPG4 GTO3 MET2 ARG1 DDR2 TPO4 YOR302W CPA1 ATG41 ARG3 YER053C-A DPI8 MNC1 YLR412C-A*

**Msn4**	Transcriptional activator related to Msn2p; activated in stress conditions, which results in translocation from the cytoplasm to the nucleus; binds DNA at stress response elements of responsive genes, inducing gene expression	76,83%	2,54%	*RTC2 CIT2 HSP30 RTN2 YDR034W-B NRG1 FMP16 DLD3 PRB1 PHM8 RGI1 MET6 HSP12 MET10 ZRT1 STR3 CUP2 YGL117W GSC2 CTT1 CLD1 MEP1 RTS3 ECM12 YHR022C RTC3 CRG1 PRM5 MET28 LYS1 INO1 PRM10 MHO1 CPA2 PIR3 MET14 GAP1 TFS1 NCW2 HMX1 BOP2 TMA10 ARG7 SPG4 GTO3 DIA1 MET2 ARG1 DDR2 BAG7 TPO4 YOR302W CPA1 FIT2 ATG41 EEB1 SUR1 YBL111C ATG8 ARG3 YER053C-A DPI8 SPO24*

Table S7. Transcription factors involved in the regulation of the up-regulated genes in response to neomycin. The table indicates for each transcription factor its function, the percentage of genes regulated in the list of neomycin-repressed genes, the percentage regulated in the overall *S. cerevisiae* genome and the name of the genes regulated by them. Only those transcription factors that show an enrichment statistically significant according to a χ2 test (*p*-values ≤ 0.05) are shown. The analysis was performed by using the tool Search for Transcription Factors at Yeastract website.

**Transcription Factor**	**Function**	**% in cluster**	**% in *S. cerevisiae* genome**	**Target ORF/Genes**
**Bas1**	Myb-related transcription factor involved in regulating basal and induced expression of genes of the purine and histidine biosynthesis pathways; also involved in regulation of meiotic recombination at specific genes	90,48%	0,63%	*CHA1 FIG2 AGA2 PRM2 ASG7 BNA2 PGU1 CDA1 P RM6 PRM1 AGA1 IZH4 PRM3 FIG1 TIP1 YJL045W Y GR109W-B YIL082W-A MIP6*
**Gln3**	Transcriptional activator of genes regulated by nitrogen catabolite repression (NCR), localization and activity regulated by quality of nitrogen source	95,24%	0,83%	*CHA1 FIG2 AGA2 PRM2 ASG7 BNA2 PGU1 CDA1 P RM6 PRM1 SNN1 AGA1 IZH4 PRM3 BNA4 FIG1 TI P1 YJL045W YIL082W-A MIP6*
**Sok2**	Nuclear protein that plays a regulatory role in the cyclic AMP (cAMP)-dependent protein kinase (PKA) signal transduction pathway; negatively regulates pseudohyphal differentiation; homologous to several transcription factors	76,19%	0,70%	*CHA1 FIG2 AGA2 PRM2 ASG7 BNA2 PGU1 PRM6 P RM1 AGA1 IZH4 PRM3 BNA4 FIG1 TIP1 YJL045W*

**Hac1**	Basic leucine zipper (bZIP) transcription factor (ATF/CREB1 homolog) that regulates the unfolded protein response, via UPRE binding, and membrane biogenesis; ER stress-induced splicing pathway facilitates efficient Hac1p synthesis	61,90%	0,89%	*CHA1 FIG2 AGA2 PRM2 ASG7 BNA2 PGU1 PRM1 AGA1 IZH4 PRM3 FIG1 TIP1*
**Spt3**	Subunit of the SAGA and SAGA-like transcriptional regulatory complexes, interacts with Spt15p to activate transcription of some RNA polymerase II-dependent genes, also functions to inhibit transcription at some promoters	47,62%	0,81%	*CHA1 AGA2 PGU1 PRM1 AGA1 IZH4 BNA4 FIG1 YGR109W-B YIL082W-A*
**Pho4**	Basic helix-loop-helix (bHLH) transcription factor of the myc-family; activates transcription cooperatively with Pho2p in response to phosphate limitation; binding to 'CACGTG' motif is regulated by chromatin restriction, competitive binding of Cbf1p to the same DNA binding motif and cooperation with Pho2p,; function is regulated by phosphorylation at multiple sites and by phosphate availability	47,62%	0,83%	*CHA1 AGA2 PRM2 ASG7 BNA2 CDA1 IZH4 BNA4 TIP1 MIP6*
**Hms1**	Basic helix-loop-helix (bHLH) protein with similarity to myc-family transcription factors; overexpression confers hyperfilamentous growth and suppresses the pseudohyphal filamentation defect of a diploid mep1 mep2 homozygous null mutant	52,38%	0,93%	*FIG2 AGA2 PRM2 ASG7 PRM6 PRM1 SNN1 AGA1 BNA4 FIG1 TIP1*
**Hap4**	Subunit of the heme-activated, glucose-repressed Hap2p/3p/4p/5p CCAAT-binding complex, a transcriptional activator and global regulator of respiratory gene expression; provides the principal activation	47,62%	0,92%	*CHA1 FIG2 AGA2 ASG7 BNA2 PRM1 AGA1 IZH4 FIG1 MIP6*

	function of the complex			
**Yox1**	Homeodomain-containing transcriptional repressor, binds to Mcm1p and to early cell cycle boxes (ECBs) in the promoters of cell cycle-regulated genes expressed in M/G1 phase; expression is cell cycle-regulated; potential Cdc28p substrate	61,90%	1,21%	*CHA1 FIG2 AGA2 PRM2 ASG7 PRM6 PRM1 AGA1 IZH4 PRM3 FIG1 TIP1 YIL082W-A*
**Pho2**	Homeobox transcription factor; regulatory targets include genes involved in phosphate metabolism; binds cooperatively with Pho4p to the PHO5 promoter; phosphorylation of Pho2p facilitates interaction with Pho4p	47,62%	0,94%	*CHA1 AGA2 PRM2 ASG7 BNA2 CDA1 PRM6 PRM1 FIG1 TIP1*
**Yhp1**	One of two homeobox transcriptional repressors (see also Yox1p), that bind to Mcm1p and to early cell cycle box (ECB) elements of cell cycle regulated genes, thereby restricting ECB-mediated transcription to the M/G1 interval	66,67%	1,44%	*CHA1 FIG2 AGA2 PRM2 CDA1 PRM6 PRM1 AGA1 IZH4 PRM3 BNA4 FIG1 TIP1 YJL045W*
**Rox1**	Heme-dependent repressor of hypoxic genes; contains an HMG domain that is responsible for DNA bending activity	47,62%	1,10%	*FIG2 PRM2 ASG7 PRM6 PRM1 AGA1 PRM3 FIG1 TIP1 YJL045W*
**Mot3**	Nuclear transcription factor with two Cys2-His2 zinc fingers; involved in repression of a subset of hypoxic genes by Rox1p, repression of several DAN/TIR genes during aerobic growth, and repression of ergosterol biosynthetic genes; can form the [MOT3+] prion	47,62%	1,13%	*CHA1 AGA2 ASG7 PRM6 PRM1 SNN1 AGA1 IZH4 FIG1 TIP1*
**Yap5**	Basic leucine zipper (bZIP) transcription factor	42,86%	1,02%	*FIG2 AGA2 PRM2 ASG7 IZH4 PRM3 BNA4 YJL045W YIL082W-A*
**Kar4**	Transcription factor required for gene regulation in response to pheromones; also required during meiosis; exists in two forms, a slower-migrating form more abundant during vegetative growth and a	57,14%	1,39%	*FIG2 AGA2 PRM2 BNA2 CDA1 PRM1 AGA1 IZH4 PRM3 FIG1 TIP1 YIL082W-A*

	faster-migrating form induced by pheromone			
**Rph1**	JmjC domain-containing histone demethylase; specifically demethylates H3K36 tri- and dimethyl modification states; associates with actively transcribed (RNA polymerase II) regions in vivo and specifically targets H3K36 in its trimethylation state as its substrate; transcriptional repressor of PHR1; Rph1p phosphorylation during DNA damage is under control of the MEC1-RAD53 pathway	38,10%	1,12%	*CHA1 AGA2 PRM2 BNA2 PGU1 PRM3 BNA4 YJL045W*
**Rlm1**	MADS-box transcription factor, component of the protein kinase C-mediated MAP kinase pathway involved in the maintenance of cell integrity; phosphorylated and activated by the MAP-kinase Slt2p	52,38%	1,55%	*PRM2 PGU1 PRM6 PRM1 AGA1 PRM3 FIG1 TIP1 YGR109W-B YIL082W-A MIP6*
**Rme1**	Zinc finger protein involved in control of meiosis; prevents meiosis by repressing IME1 expression and promotes mitosis by activating CLN2 expression; directly repressed by a1-alpha2 regulator; mediates cell type control of sporulation	47,62%	1,55%	*FIG2 AGA2 PRM2 ASG7 PRM1 AGA1 IZH4 FIG1 YJL045W MIP6*
