# Supplementary material for: Neomycin Interferes with Phosphatidylinositol-4,5-Bisphosphate at the Yeast Plasma Membrane and Activates the Cell Wall Integrity Pathway
Source: Int J Mol Sci. 2022 Sep 20;23(19):11034. doi: 10.3390/ijms231911034 (PMC9569482; doi:10.3390/ijms231911034)
Supplement: Supplementary file 1 [file ijms-23-11034-s001.zip › Table S7.pdf]

Table S7. Transcription factors involved in the regulation of the down-regulated genes in response to neomycin. The table indicates for each transcription factor its function, the percentage of genes regulated in the list of neomycin-repressed genes, the percentage regulated in the overall *S. cerevisiae* genome and the name of the genes regulated by them. Only those transcription factors that show an statistically significant enrichment according to a $\chi 2$ test (p-values ≤ 0.05) are shown. The analysis was performed by using the tool Search for Transcription Factors at Yeastract website.

**Transcription Factor**	**Function**	**% in cluster**	**% in *S. cerevisiae* genome**	**Target ORF/Genes**
**Bas1**	Myb-related transcription factor involved in regulating basal and induced expression of genes of the purine and histidine biosynthesis pathways; also involved in regulation of meiotic recombination at specific genes	90,48%	0,63%	*CHA1 FIG2 AGA2 PRM2 ASG7 BNA2 PGU1 CDA1 PRM6 PRM1 AGA1 IZH4 PRM3 FIG1 TIP1 YJL045W YGR109W-B YIL082W-A MIP6*
**Gln3**	Transcriptional activator of genes regulated by nitrogen catabolite repression (NCR), localization and activity regulated by quality of nitrogen source	95,24%	0,83%	*CHA1 FIG2 AGA2 PRM2 ASG7 BNA2 PGU1 CDA1 PRM6 PRM1 SNN1 AGA1 IZH4 PRM3 BNA4 FIG1 TIP1 YJL045W YIL082W-A MIP6*
**Sok2**	Nuclear protein that plays a regulatory role in the cyclic AMP (cAMP)-dependent protein kinase (PKA) signal transduction pathway; negatively regulates pseudohyphal differentiation; homologous to several transcription factors	76,19%	0,70%	*CHA1 FIG2 AGA2 PRM2 ASG7 BNA2 PGU1 PRM6 PRM1 AGA1 IZH4 PRM3 BNA4 FIG1 TIP1 YJL045W*
**Hac1**	Basic leucine zipper (bZIP) transcription factor (ATF/CREB1 homolog) that regulates the unfolded protein response, via UPRE binding, and membrane biogenesis; ER stress-induced splicing pathway facilitates efficient Hac1p synthesis	61,90%	0,89%	*CHA1 FIG2 AGA2 PRM2 ASG7 BNA2 PGU1 PRM1 AGA1 IZH4 PRM3 FIG1 TIP1*
**Spt3**	Subunit of the SAGA and SAGA-like transcriptional regulatory complexes, interacts with Spt15p to activate transcription of some RNA polymerase II-dependent genes, also functions to inhibit transcription at some promoters	47,62%	0,81%	*CHA1 AGA2 PGU1 PRM1 AGA1 IZH4 BNA4 FIG1 YGR109W-B YIL082W-A*

**Pho4**	Basic helix-loop-helix (bHLH) transcription factor of the myc-family; activates transcription cooperatively with Pho2p in response to phosphate limitation; binding to 'CACGTG' motif is regulated by chromatin restriction, competitive binding of Cbf1p to the same DNA binding motif and cooperation with Pho2p,; function is regulated by phosphorylation at multiple sites and by phosphate availability	47,62%	0,83%	*CHA1 AGA2 PRM2 ASG7 BNA2 CDA1 IZH4 BNA4 TIP1 MIP6*
**Hms1**	Basic helix-loop-helix (bHLH) protein with similarity to myc-family transcription factors; overexpression confers hyperfilamentous growth and suppresses the pseudohyphal filamentation defect of a diploid mep1 mep2 homozygous null mutant	52,38%	0,93%	*FIG2 AGA2 PRM2 ASG7 PRM6 PRM1 SNN1 AGA1 BNA4 FIG1 TIP1*
**Hap4**	Subunit of the heme-activated, glucose-repressed Hap2p/3p/4p/5p CCAAT-binding complex, a transcriptional activator and global regulator of respiratory gene expression; provides the principal activation function of the complex	47,62%	0,92%	*CHA1 FIG2 AGA2 ASG7 BNA2 PRM1 AGA1 IZH4 FIG1 MIP6*
**Yox1**	Homeodomain-containing transcriptional repressor, binds to Mcm1p and to early cell cycle boxes (ECBs) in the promoters of cell cycle-regulated genes expressed in M/G1 phase; expression is cell cycle-regulated; potential Cdc28p substrate	61,90%	1,21%	*CHA1 FIG2 AGA2 PRM2 ASG7 PRM6 PRM1 AGA1 IZH4 PRM3 FIG1 TIP1 YIL082W-A*
**Pho2**	Homeobox transcription factor; regulatory targets include genes involved in phosphate metabolism; binds cooperatively with Pho4p to the PHO5 promoter; phosphorylation of Pho2p facilitates interaction with Pho4p	47,62%	0,94%	*CHA1 AGA2 PRM2 ASG7 BNA2 CDA1 PRM6 PRM1 FIG1 TIP1*

**Yhp1**	One of two homeobox transcriptional repressors (see also Yox1p), that bind to Mcm1p and to early cell cycle box (ECB) elements of cell cycle regulated genes, thereby restricting ECB-mediated transcription to the M/G1 interval	66,67%	1,44%	*CHA1 FIG2 AGA2 PRM2 CDA1 PRM6 PRM1 AGA1 IZH4 PRM3 BNA4 FIG1 TIP1 YJL045W*
**Rox1**	Heme-dependent repressor of hypoxic genes; contains an HMG domain that is responsible for DNA bending activity	47,62%	1,10%	*FIG2 PRM2 ASG7 PRM6 PRM1 AGA1 PRM3 FIG1 TIP1 YJL045W*
**Mot3**	Nuclear transcription factor with two Cys2-His2 zinc fingers; involved in repression of a subset of hypoxic genes by Rox1p, repression of several DAN/TIR genes during aerobic growth, and repression of ergosterol biosynthetic genes; can form the [MOT3+] prion	47,62%	1,13%	*CHA1 AGA2 ASG7 PRM6 PRM1 SNN1 AGA1 IZH4 FIG1 TIP1*
**Yap5**	Basic leucine zipper (bZIP) transcription factor	42,86%	1,02%	*FIG2 AGA2 PRM2 ASG7 IZH4 PRM3 BNA4 YJL045W YIL082W-A*
**Kar4**	Transcription factor required for gene regulation in response to pheromones; also required during meiosis; exists in two forms, a slower-migrating form more abundant during vegetative growth and a faster-migrating form induced by pheromone	57,14%	1,39%	*FIG2 AGA2 PRM2 BNA2 CDA1 PRM1 AGA1 IZH4 PRM3 FIG1 TIP1 YIL082W-A*
**Rph1**	JmjC domain-containing histone demethylase; specifically demethylates H3K36 tri- and dimethyl modification states; associates with actively transcribed (RNA polymerase II) regions in vivo and specifically targets H3K36 in its trimethylation state as its substrate; transcriptional repressor of PHR1; Rph1p phosphorylation during DNA damage is under control of the MEC1-RAD53 pathway	38,10%	1,12%	*CHA1 AGA2 PRM2 BNA2 PGU1 PRM3 BNA4 YJL045W*

**Rlm1**	MADS-box transcription factor, component of the protein kinase C-mediated MAP kinase pathway involved in the maintenance of cell integrity; phosphorylated and activated by the MAP-kinase Slt2p	52,38%	1,55%	*PRM2 PGU1 PRM6 PRM1 AGA1 PRM3 FIG1 TIP1 YGR109W-B YIL082W-A MIP6*
**Rme1**	Zinc finger protein involved in control of meiosis; prevents meiosis by repressing IME1 expression and promotes mitosis by activating CLN2 expression; directly repressed by a1-alpha2 regulator; mediates cell type control of sporulation	47,62%	1,55%	*FIG2 AGA2 PRM2 ASG7 PRM1 AGA1 IZH4 FIG1 YJL045W MIP6*
