# Supplementary material for: Neomycin Interferes with Phosphatidylinositol-4,5-Bisphosphate at the Yeast Plasma Membrane and Activates the Cell Wall Integrity Pathway
Source: Int J Mol Sci. 2022 Sep 20;23(19):11034. doi: 10.3390/ijms231911034 (PMC9569482; doi:10.3390/ijms231911034)
Supplement: Supplementary file 1 [file ijms-23-11034-s001.zip › Table S8.pdf]

Table S8. List of strains used and generated throughout this work. The table indicates the name of the strain, its genotype and the reference.

Strain	Genotype	Reference
**BY4741**	*MATa; his3Δ1; leu2Δ0; met15Δ0; ura3Δ0*	Euroscarf, Germany
**BY4741 RLM1-6MYC**	BY4741; *RLM1-6MYC::URA3*	This study
**Y3656**	*MATα; can1Δ::MFA1pr-HIS3-MFα1pr-LEU2; his3Δ1; leu2Δ0; ura3Δ0; met15Δ0; lys2Δ0*	[73]
**YSTH1**	Y3656; *HO::natMX6::SSB1*	This study
**YSTH2**	Y3656; *HO::MLP1p-MKK1*^{S386P}*-ADHt-natMX6::SSB1*	This study
slt2Δ	Y3656; *slt2Δ::natMX6*	[28]
wsc1Δ	Y3656; *wsc1Δ::natMX6*	[28]
mid2Δ	Y3656; *mid2Δ::natMX6*	[28]
msg5Δ	Y3656; *msg5Δ::natMX6*	[28]
rlm1Δ	Y3656; *rlm1Δ::natMX6*	[28]
bck1Δ	Y3656; *bck1Δ::natMX6*	[28]
rom2Δ	Y3656; *rom2Δ::natMX6*	[28]
mkk1Δ	Y3656; *mkk1Δ::natMX6*	[28]
mkk2Δ	Y3656; *mkk2Δ::natMX6*	[28]
swi4Δ	Y3656; *swi4Δ::natMX6*	[28]
swi6Δ	Y3656; *swi6Δ::natMX6*	[28]
ptc1Δ	Y3656; *ptc1Δ::natMX6*	[28]
**CML128**	*MATa; leu2Δ3,112; ura3Δ52; trp1Δ; his4Δ; can1*^{r}	[74]
pkc1Δ	CML128; *pkc1Δ*::LEU2	[75]
**YPH499**	*MATa ade2-101 trp1-63 leu2-1 ura3-52 his3-200 lys2-801*	[76]

References

28. Martin, H.; Shales, M.; Fernandez-Piñar, P.; Wei, P.; Molina, M.; Fiedler, D.; Shokat, K. M.; Beltrao, P.; Lim, W.; Krogan, N. J. Differential Genetic Interactions of Yeast Stress Response MAPK Pathways. *Mol. Syst. Biol.* **2015**, *11* (4), 800.

73. Schuldiner, M.; Collins, S. R.; Thompson, N. J.; Denic, V.; Bhamidipati, A.; Punna, T.; Ihmels, J.; Andrews, B.; Boone, C.; Greenblatt, J. F.; Weissman, J. S.; Krogan, N. J. Exploration of the Function and Organization of the Yeast Early Secretory Pathway through an Epistatic Miniarray Profile. *Cell* **2005**, *123* (3), 507–519.

74. Gallego, C.; Garí, E.; Colomina, N.; Herrero, E.; Aldea, M. The Cln3 Cyclin Is Down-Regulated by Translational Repression and Degradation during the G1 Arrest Caused by Nitrogen Deprivation in Budding Yeast. *EMBO J.* **1997**, *16* (23), 7196–7206.

75. De La Torre-Ruiz, M. A.; Torres, J.; Ariño, J.; Herrero, E. Sit4 Is Required for Proper Modulation of the Biological Functions Mediated by Pkc1 and the Cell Integrity Pathway in *Saccharomyces cerevisiae*. *J. Biol. Chem.* **2002**, *277* (36), 33468–33476.

76. Sikorski, R. S.; Hieter, P. A System of Shuttle Vectors and Yeast Host Strains Designed for Efficient Manipulation of DNA in Saccharomyces Cerevisiae. *Genetics* **1989**, *122* (1), 19–27.
